# Exploring the association between multi-dimensional poverty and antibiotic resistance: findings from a mixed-methods study in Pakistan

**DOI:** 10.1016/j.lansea.2025.100656

**Published:** 2025-08-27

**Authors:** Iltaf Hussain, Muhammad Fawad Rasool, Jamshid Ullah, Muhammad Nafees, Inzemam Khan, Muhtar Kadirhaz, Miaomiao Xu, Chengzhou Tang, Yi Dong, Wei Zhao, Jie Chang, Yu Fang

**Affiliations:** a Department of Pharmacy Administration, School of Pharmacy, Xi'an Jiaotong University, Xi'an, China; b Center for Drug Safety and Policy Research, Xi'an Jiaotong University, Xi'an, China; c Shaanxi Center for Health Reform and Development Research, Xi'an, China; d Department of Pharmacy Practice, Faculty of Pharmacy, Bahauddin Zakariya University, Multan, Pakistan; e Institute of Paramedical Sciences, Khyber Medical University, Peshawar, Pakistan; f Department of Pharmacy, University of Peshawar, Peshawar, Pakistan

**Keywords:** Poverty, Antibiotic resistance, WASH practices, Antibiotic misuse

## Abstract

**Background:**

Poverty is a potential contributor to antibiotic resistance; however, the previous studies have not adequately addressed the role of poverty in shaping antibiotic resistance through social inequalities. Considering this, the current study evaluated the role of multi-dimensional poverty in antibiotic resistance.

**Methods:**

A mixed-method study was conducted in three provinces of Pakistan using multistage sampling to recruit physician-confirmed urinary tract infection (UTI) patients from public laboratories. Antibiotic resistance data were collected from susceptibility reports, while poverty was measured using the multi-dimensional poverty index (MPI). Water, sanitation and hygiene (WASH) practices were assessed through a self-developed, validated questionnaire. Survey-weighted logistic regression analysis examined the association between MPI and antibiotic resistance.

**Findings:**

A total of 698 patients were recruited, with more than half being in some level of deprivation (total = 413, vulnerable: 117, deprived: 76, severely deprived: 220). Multidimensional poverty was independently associated with increased odds of multidrug resistance (MDR). The risk of MDR was significantly increase across the deprivation level in unadjusted analysis (vulnerable; OR: 1.94, 95% CI 1.11–3.39, deprived; OR: 2.05, 95% CI 1.06–3.98, and severely deprived: OR: 1.80, 95% CI 1.04–3.09). After adjusting for antibiotics misuse and poor WASH practices, the association persisted. In the fully adjusted model, the risk of MDR was further increased in the poorer-subgroups, (vulnerable; aORs: 3.03, 95% CI 1.33–6.73, deprived; aOR: 3.01, 95% CI 1.26–7.15, and severely deprived; aOR: 4.28 95% CI 1.74–10.49). The qualitative interviews (n = 34) from patients highlighted that financial barriers drove self-medication with leftover antibiotics and treatment non-adherence. Poor WASH infrastructure was described as a systemic contributor to infection spread. In addition, patients in the poorer subgroups were presented with delayed treatment seeking.

**Interpretation:**

The risk of antibiotic resistance increases with the increasing levels of deprivation; however, we should not assume that higher deprivation directly drives antibiotic resistance. Instead, structural barriers such as limited healthcare access, poor WASH infrastructure, and financial constraints create an environment where self-medication, treatment non-adherence, and infection transmission occur across all poverty levels, not just because of individual choices. These findings emphasize the need for interventions that address healthcare inequities, improve WASH infrastructure, and regulate antibiotic access, combined with behavior-changing interventions.

**Funding:**

This work was funded by the “Young Talent Support Plan” of the Health Science Center, 10.13039/501100002412Xi’an Jiaotong University, and the 10.13039/501100001809National Natural Science Foundation of China (grant number 72274150).

**Research in context**

**Evidence before this study**

We searched PubMed, Embase, and the Web of Science using the terms “poverty”, “multi-dimensional poverty”, “antibacterial”, “antimicrobial”, “antibiotic”, “AMR”, “antimicrobial resistance”, “antibiotic resistance”, “socioeconomic” and “socio∗ risk factors” for English articles published between January 2010 and May 2024.

Regarding antibiotic resistance, we identified two reviews (published in 2018 and 2022, respectively) that covered 62 articles on the association between poverty and antibiotic resistance. However, one review was limited to the Canadian context, and the other review used the dimension of poverty, including living conditions, water and sanitation, education, and social deprivation, and reported inconsistent associations. While most studies from low- and middle-income countries (LMIC) found a negative association of living conditions and income with antibiotic resistance; studies from high-income countries (HIC), in contrast, reported a positive association. Meanwhile, water and sanitation were positively associated with antibiotic resistance, as reported by studies from the LMICs.

Moreover, previous studies on poverty and antibiotic resistance have inadequately addressed the role of poverty in antibiotic resistance within the context of social inequalities in developing countries. Poverty measurements in these studies vary widely, often focusing on single dimensions like income or living standards, overlooking the interconnectedness of poverty indicators. There remains a lack of comprehensive analysis that captures the intricate interplay between poverty and antibiotic resistance.

**Added value of this study**

Rather than focusing on single dimensions of poverty, such as income or living standards, our study employed the internationally recognized Multidimensional Poverty Index (MPI) to capture a holistic view of poverty. This index accounts for multiple dimensions of well-being, including education, health, and living standards. Using a mixed-method approach, we integrated quantitative data from individual patients' resistance profiles with qualitative evidence from in-depth interviews. Our study was conducted across three provinces out of four in Pakistan.

Our findings revealed that individuals experiencing higher levels of deprivation were more likely to exhibit multidrug resistance (MDR). This association was influenced by various factors, including antibiotic misuse and poor water, sanitation, and hygiene practices. The qualitative data provided deeper insights into the lived experiences of deprived individuals, highlighting how financial constraints, limited access to healthcare, and inadequate sanitation contribute to antibiotic resistance.

**Implications of all the available evidence**

Understanding the contextual and situational factors of antibiotic misuse and resistance is contingent on exploring the multidimensional nature of poverty. MDR development due to self-medication and incomplete courses of antibiotics is a highly critical issue, as financial constraints prevent some patients from obtaining adequate treatment. In deprived communities, the transmission of resistant bacteria is also increased by inadequate practices. Our findings emphasize the need for comprehensive strategies that address structural barriers preventing optimal antibiotic use in LMICs. These strategies should focus on improving access to healthcare, enhancing education about antibiotic use, and ensuring adequate sanitation facilities. By addressing the root causes of poverty and improving public health infrastructure, we can mitigate the impact of antibiotic resistance and improve health outcomes in LMICs.

## Introduction

Antibiotic resistance, a complex biosocial phenomenon, threatens the potency of antibiotics, which saves millions of lives.^1^ It has become a silent pandemic and a critical threat to global public health. In 2021, antibiotic resistance was associated with 4.71 million deaths globally, whereas the forecasted deaths in 2050 were 8.2 million.^2^ The widespread inappropriate use of antibiotics is one of the potential drivers that exacerbates antibiotic resistance.^1^ Between 2000 and 2015, global human antibiotic consumption increased by over 60%, with a remarkably rapid rise observed in low- and middle-income countries (LMICs).^3^

Multi-dimension poverty is a multifaceted construct that extends beyond mere income deprivation to include overlapping deprivation in education, health, living standards, and access to essential services.^4^ Recent estimates from Pakistan indicate that 33.4% of the population experiences severe multidimensional poverty, with disproportionate impacts on access to clean water, sanitation, and healthcare.^5^ Previous studies suggested that poverty can play a significant role in the development and spread of antibiotic resistance through structural socio-economic factors.^6^^,^^7^ Poor sanitation increases the risk of infection and mediates the spread of resistant bacteria.^8^ A recent systematic review argued that poor social conditions, like homelessness, were significantly associated with the spread of resistant infections.^6^ Furthermore, under-resourced healthcare systems, coupled with weak regulatory policies for antibiotic dispensing, foster an environment favorable to antibiotic misuse. This includes both over-prescription by healthcare providers and the unregulated sale of antibiotics, which exacerbates resistance development.^9^ A recent study from Africa assessed the role of multi-dimensional poverty in antibiotic misuse in the context of social inequalities and suggested that structural barriers like inefficiencies in public healthcare, along with time and financial constraints, drive antibiotic misuse irrespective of the deprivation levels.^9^ Although, this study provides a valuable insight into how poverty shapes antibiotic misuse behaviors, it does not assess the association between multi-dimension poverty and antibiotic resistance.

While some previous studies have assessed the association between poverty and antibiotic resistance, there remains a lack of studies systematically integrated standardized, patients level multi-dimension poverty with antibiotic resistance in developing countries.^6^^,^^10^ In addition, the measurement of poverty in these studies varies, ranging from broad definitions that include economic indicators and social determinants of health to specific metrics like income.^6^^,^^10^ Many of these studies focus on single dimensions of poverty, such as income or living standards, which may overlook the interconnectedness of various poverty indicators and their collective influence on antibiotic resistance. There remains a lack of comprehensive analysis that captures the intricate interplay between poverty and antibiotic resistance.

**Fig. 1:**
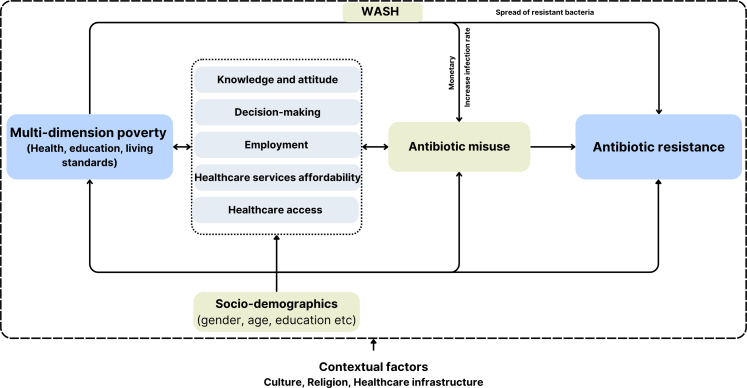
**Evidenced-based conceptual framework of multi-dimensional poverty and antibiotic resistance for the current study**.

To address above-mentioned research gaps, we conducted the current mixed-method study to assess the association between multi-dimensional poverty, guided by the evidence-based conceptual framework that explains the complex interplay of social, cultural, and economic factors in the association between poverty and antibiotic resistance, as illustrated in Fig. 1. Multi-dimensional poverty refers to the deprivation of basic human capabilities, captured through overlapping non-monetary deprivation in health, education and standard of living, as measured by Multidimensional Poverty Index (MPI). However, to cover the monetary dimension, we assessed the healthcare affordability of the patients, as financial constraints can directly contribute to treatment non-adherence, either by limiting the ability to afford complete treatment courses or by encouraging self-medication due to the unaffordability of formal healthcare.^7^^,^^11^ Poor sanitation can increase susceptibility to infectious diseases and increase the need for antibiotics. Additionally, it increases the risk of exposure to resistant bacteria.^12^^,^^13^ Contextual, attitudinal, and structural factors may mediate the relationship between poverty and antibiotic resistance. For instance, educational level can shape antibiotic knowledge, potentially leading to misuse and, thus, antibiotic resistance.^14^, ^15^, ^16^ Furthermore, the association between poverty and antibiotic resistance may be shaped by sociodemographic, cultural, religious, and contextual factors, including the disease and pathogen environment, pluralistic healthcare systems, and the effectiveness of regulatory frameworks.^17^

## Methods

### Study design and duration

Our mixed-method study design was conducted to explore the role of multi-dimensional poverty in antibiotic resistance in three provinces of Pakistan. The data integration followed the sequential explanatory design, with qualitative findings contextualizing quantitative findings. The duration of the current study was from March to November 2024. The quantitative part of the study was reported according to the Strengthening the Reporting of Observational Studies in Epidemiology (STROBE) Guidelines.^18^

### Quantitative part

Firstly, we selected three provinces (Punjab, Khyber Pakhtunkhwa, and Balochistan) out of four provinces based on the Pakistan Social and Living Standards Measurement Survey (PSLM) 2019–20 and Pakistan Economic Survey (PES) 2022–23. The selected three provinces were stratified into high (Punjab), Medium (Khyber Pakhtunkhwa), and low (Balochistan) socioeconomic stratums. In the second step, we identified the tertiary care hospitals (hereafter “hospitals”) in each province through the respective health directorate in each province and the Pakistan Bureau of Statistics. The patients were proportionally allocated to each province based on the number of hospitals (Punjab = 357, Khyber Pakhtunkhwa = 214, and Balochistan = 118). In the third step, the laboratories (hereafter “labs”) near the hospitals were treated as clusters and randomly selected in each province. Two labs from each cluster were selected, and the patients were distributed among the labs. At each selected lab, patients meeting the inclusion criteria were conveniently selected. The details regarding the sampling strategy are given in Supplementary Figure S1.

The patients of the current study were physician-confirmed UTI patients. The patients who were intended for the urine culture with an antimicrobial susceptibility test were initially selected from the labs. The selected patient was then objectively confirmed by the data collector, who assessed UTI-like symptoms and confirmed uropathogen (*E. coli*, Klebsiella spp.) in the lab report. The selected patient was further subjectively confirmed by the physicians. The details are given in Supplementary Figure S2.

The data were collected using trained data collectors (Total = 13, Punjab = 5, Kpk = 5, and Balochistan = 3). The data were collected using Microsoft forms®. The questionnaires were incorporated into Microsoft Forms® and shared with data collectors. Unique codes were assigned to each data collector. Monthly meetings were conducted with data collectors to ensure data validation and its process.

The initial draft of the questionnaire was developed based on the previous literature.^6^^,^^9^^,^^19^, ^20^, ^21^ The initially developed draft was face-validated by three experts in the field (academic = 2, physician = 1). In addition, a pilot study (n = 68) was conducted to assess internal consistency. The Cronbach alpha value of the overall questionnaire was 0.79, indicating good internal consistency. The participants included in the pilot phase were excluded from the final analysis. The details of the reliability analysis are given in Supplementary Table S1.

The study questionnaire was composed of five sections. The first section was related to demographic data, including study area, gender, age, marital status, family structure, number of people living in a home, education level, employment status, history of UTI, and ability to meet healthcare costs. Multi-dimensional poverty was measured using an MPI.^9^ The patient's pathways were also assessed to identify the treatment-seeking behavior (Supplementary Table S2). Antibiotic misuse was measured using three dichotomous questions, including self-medication, course completion, and dose skipping (Supplementary Table S3). The water, sanitation, and hygiene (WASH) practices were measured using the adopted WASH questionnaire from the United Nations High Commissioner for Refugees (UNHCR) and the World Health Organization (WHO).^20^^,^^21^ The WASH practices questionnaire was composed of five sub-sections, including hand washing, drinking water, toilet/latrine, and trash disposal, animal ownership, and use of milk (Supplementary Table S4).

The antibiotic susceptibility data were collected from the culture sensitivity report of the included patients. The data were documented in a pre-defined worksheet for categorizing isolates of Enterobacteriaceae.^22^ A pre-defined criterion was used to define multidrug resistance (MDR, non-susceptible to ≥1 agent in >3 antimicrobial categories). In addition, we also used the World Health Organization (WHO) Access, Watch, and Reserve (AWaRe) classification to assess the distribution of resistance across AWaRe categories. The details are given in Supplementary Table S5.

Descriptive statistics were employed to summarize the sociodemographic characteristics, antibiotic use behaviors, WASH practices, and MDR among participants. Frequencies and proportions were reported for categorical variables, whereas means and standard deviations were calculated for continuous variables.

To evaluate the relationship between the MPI and MDR, we carried out survey-weighted logistic regression analyses. We first fitted unadjusted models to observe the crude associations between MPI categories and MDR. Then, a stepwise approach was used to assess the impact of potential confounders by adding one covariate at a time, such as antibiotic misuse (self-medication, course completion, dose skipping), WASH practices, gender, age, marital status, education, employment, number of family members, ability to afford healthcare, symptom-related stigma, and healthcare advice. Finally, a multivariable survey-weighted logistic regression model was developed, adjusting for all covariates. Results are reported as odds ratios (ORs), adjusted odds ratios (aORs), 95% confidence intervals (CIs), and robust standard errors.

All analyses were conducted using R statistical software (version 4.5.1). A two-sided *p*-value equal or less than 0.05 was considered statistically significant.

#### Qualitative part

A semi-structured interview guide was developed based on the previously published literature.^9^^,^^23^^,^^24^ In addition, the interview guide was further guided by the Social Determinants of Health (SDH) framework^25^ and the Health Belief Model (HBM).^26^ The SDH framework conceptualizes that various social, economic, and environmental factors influence health. It aided the interview guide in exploring how poverty-related factors (like access to healthcare, education, living conditions, and economic stability) impact antibiotic use and resistance. HBM explains that people's health-related behavior can be predicted by their beliefs about health problems/conditions, perceived benefits of action, and barriers to the action. Therefore, HBM guided the interview questions about participants' understanding of antibiotics and antimicrobial resistance, perceived risks and benefits, and barriers to proper antibiotic use. The interview guide comprised open-ended questions regarding participant knowledge about antibiotics and antibiotic resistance, access to healthcare services and medication, living conditions and sanitation, and socio-cultural factors associated with the treatment of the disease. The details of the interview guide can be seen in Supplementary Table S6. Four potential patients, who were excluded from the final analysis, were subjected to a pilot study to assess their understanding of the questions.

Using a purposive subsample of the quantitative cohort, we conducted interviews with physician-confirmed UTI patients who had completed the survey and were willing to participate in individual interviews. At each selected laboratory, trained local data collectors approached patients immediately after the quantitative data collection and explained the objectives of the qualitative study. The interviews were conducted and recorded in the native language by trained local interviewers. The interviews were then transcribed and translated into English by two authors (IH, MFR) who were fluent in both languages.

We analyzed qualitative data obtained from in-depth interviews employing thematic analysis, specifically Braun and Clarke's reflexive thematic analysis,^27^ selected for its flexibility and capacity to effectively capture participants' lived experiences concerning multidimensional poverty and antibiotic resistance. The analysis was primarily inductive, with themes emerging directly from the data rather than being constrained by pre-existing categories. Although the interview guide was developed based on the HBM and the SDH framework to ensure comprehensive coverage of relevant topics, the analysis itself remained inductive, facilitating a participant-driven exploration of the data. The process encompassed multiple readings of the transcripts, initial coding of recurrent ideas and concepts, and iterative refinement of themes utilizing NVivo 12.

### Study ethics

The study was reviewed and approved by the Ethical Committee of Xi'an Jiaotong University, which waived the requirement for an approval number and by the Department of Pharmacy Practice, Bahauddin Zakariya University, Multan, Pakistan (Ex03-ERCPP-BZU-24). Written and verbally informed consent was taken from each patient. The patient's data was kept confidential throughout the study period.

### Role of the funding source

The funders of the study had no role in study design, data collection, data analysis, data interpretation, or writing of the report.

## Results

A total of 803 patients met the eligibility criteria; of these, 698 patients provided informed consent and participated in the current study (response rate: 87%). Among the included patients more than half were female (56.8%). Most of the study participants were younger age (18–30 years: 31%), married (75.5%), had a joint family structure (61.7%) and had no formal education (31.8%). Most of the participants were unemployed (68.1%) and had difficulty in meeting the healthcare cost (74.7%). The details are given in Table 1. The distribution of demographic characteristics across the study area is given in Supplementary Table S7. The patients with linked qualitative and quantitative data are given in Table 2.

**Table 1:** Distribution of the demographics across the multi-dimension poverty.

	Multi-dimensional poverty				
	Overall n = 698	Not deprived n = 285	Vulnerable n = 117	Deprived n = 76	Severely deprived n = 220
**Gender**					
Male	301 (43.1)	152 (50.5)	28 (9.3)	34 (11.3)	87 (28.9)
Female	397 (56.8)	133 (33.5)	89 (22.4)	42 (10.6)	133 (33.5)
**Age**					
18–30	217 (31.0)	135 (62.2)	39 (18.0)	28 (12.9)	15 (6.9)
31–40	168 (24.0)	85 (50.6)	39 (23.2)	20 (11.9)	24 (14.3)
41–50	146 (20.9)	43 (29.5)	29 (19.9)	18 (12.3)	56 (38.4)
51–60	82 (11.7)	18 (22.0)	5 (6.1)	5 (6.1)	54 (65.9)
>60	85 (12.2)	4 (4.7)	5 (5.9)	5 (5.9)	71 (83.5)
**Marital status**					
Single	144 (20.6)	91 (63.2)	26 (18.1)	20 (13.9)	7 (4.9)
Married	528 (75.5)	194 (36.7)	88 (16.7)	55 (10.4)	191 (36.2)
Other	26 (3.7)	0 (0.0)	3 (11.5)	1 (3.8)	22 (84.6)
**Family structure**					
Nuclear family	267 (38.2)	198 (74.2)	41 (15.4)	19 (7.1)	9 (3.4)
Joint/extended family	431 (61.7)	87 (20.2)	76 (17.6)	57 (13.2)	211 (49.0)
**Number of family members**					
1–2	119 (17.0)	101 (84.9)	13 (10.9)	1 (0.8)	4 (3.4)
3–6	210 (30.0)	121 (57.6)	31 (14.8)	29 (13.8)	29 (13.8)
>6	369 (52.8)	63 (17.1)	73 (19.8)	46 (12.5)	187 (50.7)
**Education**					
No education	222 (31.8)	6 (2.7)	42 (18.9)	33 (14.9)	141 (63.5)
Primary	172 (24.6)	8 (4.7)	53 (30.8)	40 (23.3)	71 (41.3)
Secondary	142 (20.3)	118 (83.1)	15 (10.6)	3 (2.1)	6 (4.2)
>Secondary	162 (23.2)	153 (94.4)	7 (4.3)	0 (0.0)	2 (1.2)
**Employment**					
Not employed	476 (68.1)	123 (25.8)	86 (18.1)	66 (13.9)	201 (42.2)
Employed	222 (31.8)	162 (73.0)	31 (14.0)	10 (4.5)	19 (8.6)
**Ability to meet healthcare cost**					
Easy	178 (25.5)	145 (81.5)	29 (16.3)	4 (2.2)	0 (0.0)
Little difficult	301 (43.1)	125 (41.5)	73 (24.3)	43 (14.3)	60 (19.9)
Very difficult	219 (31.3)	15 (6.8)	15 (6.8)	29 (13.2)	160 (73.1)
**UTI history**					
No	305 (43.6)	197 (64.6)	57 (18.7)	28 (9.2)	23 (7.5)
Yes	393 (56.2)	88 (22.4)	60 (15.3)	48 (12.2)	197 (50.1)
**Symptom stigma**					
No	437 (62.5)	243 (55.6)	65 (14.9)	41 (9.4)	88 (20.1)
Yes	261 (37.3)	42 (16.1)	52 (19.9)	35 (13.4)	132 (50.6)
**Source of healthcare advice**					
Formal	369 (52.8)	247 (66.9)	77 (20.9)	21 (5.7)	24 (6.5)
Informal	329 (47.1)	38 (11.6)	40 (12.2)	55 (16.7)	196 (59.6)
**Province**					
Punjab	357 (51.1)	160 (44.8)	50 (14.0)	45 (12.6)	102 (28.6)
Kpk	221 (31.6)	91 (41.2)	44 (19.9)	17 (7.7)	69 (31.2)
Balochistan	120 (17.2)	34 (28.3)	23 (19.2)	14 (11.7)	49 (40.8)

Kpk—Khyber Pakhtunkhwa, data was presented as: n (%).

**Table 2:** Patients’ characteristics with linked qualitative and quantitative data in the current study.

	Total (n = 34)	Punjab (n = 16)	Kpk (n = 10)	Balochistan (n = 8)
**Gender**				
Male	20 (58.8)	9 (56.3)	2 (20)	3 (37.5)
Female	14 (41.1)	7 (43.8)	8 (80)	5 (62.5)
**Age**				
18–30	15 (44.1)	7 (43.8)	5 (50)	3 (37.5)
31–40	10 (29.4)	6 (37.5)	4 (40)	0 (0)
41–50	7 (20.6)	3 (18.8)	0 (0)	4 (50)
51–60	1 (2.9)	0 (0)	0 (0)	1 (12.5)
>60	1 (2.9)	0 (0)	1 (10)	0 (0)
**Poverty status**				
Not deprived	15 (44.1)	10 (62.5)	4 (40)	1 (12.5)
Vulnerable to poverty	7 (20.6)	1 (6.3)	4 (40)	2 (25)
Deprived	5 (14.7)	2 (12.5)	1 (10)	2 (25)
Severely deprived	7 (20.6)	3 (18.8)	1 (10)	3 (37.5)

Kpk—Khyber Pakhtunkhwa, Data presented as: n (%).

**Fig. 2:**
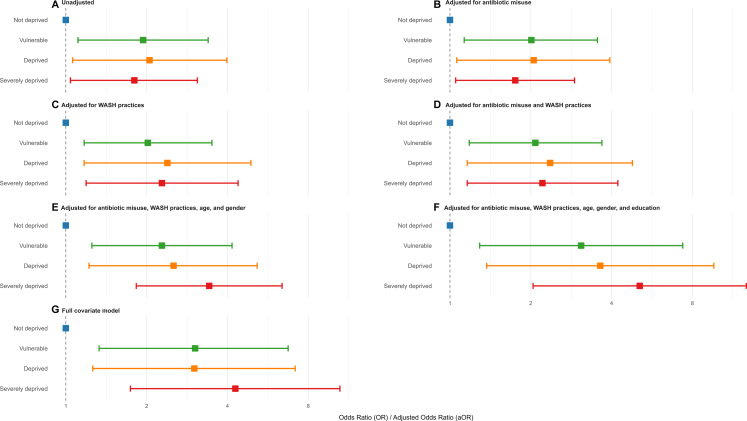
**Association of the multi-dimensional poverty with MDR**. The full covariate model was adjusted for socio-economic factors including age, gender, education, family structure, number of family members, ability to meet healthcare cost, symptoms stigma and UTI history.

Multidimensional poverty demonstrates a significant association with MDR. Patients experiencing multi-dimension poverty had significantly higher odds of MDR in unadjusted analysis (vulnerable; OR: 1.94, 95% CI 1.11–3.39; *p* = 0.02, deprived; OR 2.05, 95% CI 1.06–3.98; *p* = 0.03, and severely deprived; OR: 1.80, 95% CI 1.04–3.09; *p* = 0.03) as shown in Fig. 2A; Supplementary Table S10. The association between multidimensional poverty and MDR persisted across models adjusting for antibiotic misuse behaviors and WASH practices. The severely deprived patients had twofold higher risk of MDR (aOR 2.28, 95% CI 1.19–4.38) when adjusted for WASH practices (Fig. 2C, Supplementary Table S15). The risk of MDR remains persisted significantly when adjusted for antibiotic misuse (Fig. 2B, Supplementary Table S14). When adjusted for demographic variables including age, gender, and education the risk of MDR was significantly increased across the poverty levels (Vulnerable; aOR: 2.80, 95% CI 1.10–7.15, deprived; aOR: 3.17, 95% CI 1.12–8.92, severely deprived; aOR: 4.14, 95% CI 1.49–11.46) (Fig. 2E–G, Supplementary Table S17–S26).

Table 3 presents the themes identified from patient interviews based on their responses. Financial constraint was a rationale for delayed seeking behavior in severely deprived patients. The patients in the severely deprived level were vocal about the unaffordability of the treatment.

*“If I don’t have enough money, I wait until I can afford the medicine. Enduring pain or discomfort is sometimes hard, especially when family members are unwell. But we have no choice; we must manage and wait until we can gather enough money to buy the medicine.*” (Female, severely deprived, Khyber Pakhtunkhwa, interviewer: female)

**Table 3:** The patient's interviewees reported challenges regarding the rational use of antibiotics and good WASH practices.

Theme	Quote (s)
Financial constraints and healthcare access	*“If we don't have money, I just try to use our old medicine [antibiotics] at home. Sometimes, It's not ideal, but it's the only option available to me, especially when the pain or symptoms become unbearable, and I can not afford to go to the clinic or buy new medicine”* **Female, severely deprived, Balochistan, Interviewer: Female**
Financial constraints and antibiotic misuse	*“I use leftover antibiotics when I can not get a prescription. Sometimes, I feel the symptoms of burning urination, pain, and discomfort coming back, and I don't have money or time to see a doctor, so I just take whatever I have left at home. Its not the best option, but when you are in pain and can't afford the proper treatment, it feels like the only choice.”* **Female, Deprived, Balochistan, Interviewer: Female**
Familiarity and antibiotic misuse	*“I use what I have at home [antibiotics]. It's easier and quicker, and I have used these medicines before, so I know they might help.”* **Male, Not deprived, Khyber Pakhtunkhwa, Interviewer: Male**
Limited knowledge about antibiotics and its resistance	*“I know antibiotics are for infection, but I don't understand why sometimes they stop working.”* **Male, Not deprived, Punjab, Interviewer: Female**
Poor WASH practices	*“We don't have a proper toilet, just an open space outside. It's the basic setup where we have to go when needed. There is no privacy or proper waste management, and it's challenging to use during rainy weather.”* **Male, Severely deprived, Balochistan, Interviewer: Male** *“We wash our hands when we can, but water is a big problem. We don't always have enough water to wash our hands properly. Sometimes, we have to ration it for other needs, like cooking and drinking. It makes it hard to maintain proper hygiene consistently.* **Male, vulnerable to deprivation, Khyber Pakhtunkhwa, Interviewer: Male**

Additionally, financial constraints were found to be a significant barrier to healthcare access and a facilitator of antibiotic misuse, especially self-medication and incompletion in the poorer subgroup.

*“We can’t afford to go to the clinic, so we just try to manage at home. We often rely on home remedies or leftover medicines [antibiotics] when someone is sick, hoping they’ll help. It’s difficult because we know professional care would be better, but the costs for travel and treatment make it impossible for us to seek proper medical help*” (Female, deprived, Balochistan, interviewer: female)

*“If I feel better after a few days, I stop taking the antibiotics. Sometimes, I feel like the infection has gone away, so I think it’s okay to stop. But I also know it’s not the right thing to do, and I feel guilty because I know the doctor said I should finish the full course. But it’s hard when you don’t have enough money to buy the antibiotics full course.*” (Female, severely deprived, Punjab, interviewer: male)

Familiarity with and easy access to left-over antibiotics contributed to self-medication

*“I use what I have at home [antibiotics]. It’s easier and quicker, and I’ve used these medicines before, so I know they might help.*” (Male, not deprived, Khyber Pakhtunkhwa, interviewer: male)

Poor WASH practices in deprived groups provided the rationale for the association between multi-dimensional poverty and antibiotic resistance. Un/limited water availability in deprived groups is associated with poor WASH practices. Limited access to clean drinking water, lack of proper sanitation facilities, and insufficient water for hygiene practices create an environment conducive to spreading infections.

*“We wash our hands when we can, but water is a big problem. We don’t always have enough water to wash our hands properly, and sometimes we must ration it for other needs like cooking and drinking. It makes it hard to maintain proper hygiene consistently*” (Male, vulnerable to deprivation, Khyber Pakhtunkhwa, interviewer: male)

*“We don’t have a proper toilet, just an open space outside. It's a basic setup where we have to go when needed, and it's exposed to the elements. There’s no privacy or proper waste management, and it’s challenging to use during rainy weather.*” (Male, severely deprived, Balochistan, interviewer: male)

Individuals lack an understanding of the risks associated with irrational use, such as developing resistance, which may encourage them to use antibiotics irrationally.

*“I’ve heard that taking too many antibiotics can make them not work later, but I’m not sure why.”* (Female, vulnerable to deprivation, Balochistan, interviewer: female)

## Discussion

The current study highlighted a significant association between multi-dimensional poverty and antibiotic resistance. This association remains robust even after accounting for antibiotic misuse, WASH practices, and socio-demographic factors, highlighting the direct role of poverty in antibiotic resistance. These factors exacerbated the risk but did not fully explain the association, indicating that multidimensional poverty independently contributes to antimicrobial resistance through intersecting pathways. Qualitative insights revealed that financial constraints caused delayed treatment-seeking, leading patients toward informal healthcare sources or leftover antibiotics, which further increased the risk of antibiotic misuse. Poor WASH practices and conditions in deprived communities further amplify infection transmission, creating an environment conducive to antibiotic overuse and resistance development. Household overcrowding and limited healthcare affordability consistently sustain MDR risks.

Traditionally, income levels have been the cornerstone to measure poverty. According to the World Bank, globally, approximately 838 million people (10.5%) live in extreme poverty, with South Asia accounting for 141.5 million (7.7%) in 2024. In Pakistan, an estimated population lived in extreme poverty was 16.52% in 2018. However, after raising the international poverty line to $4.20 per day, data shows that 44.7% of Pakistan's population lives below the poverty threshold, highlighting the increasing severity of the economic deprivation in the country.^28^ However, solely relying on income overlooks the complexity and multi-dimensional nature of poverty. The development of MPI in 2011 provides a comprehensive framework by incorporating indicators across education, health, and living standards, which gives a holistic view of poverty and better reflects the diversity of challenges faced by the poor.^29^ Global MPI was adopted previously by Green et al. with reduced indicators to measure the poverty status in African countries, justified by the focus on the adult population and data availability.^9^ Similarly, we used the same adopted MPI in the current study, as we also focused on the adult population and faced limited data availability on other indicators of the global MPI.

It has been suggested previously that poverty plays a significant role in antibiotic resistance. Studies indicate that socioeconomic deprivation plays an important role in the development of antibiotic resistance.^7^^,^^11^ However, most studies focus on income as a primary measure of poverty, with mixed findings. Some studies argued that income level is negatively associated with antibiotic resistance, while some reported a positive association.^6^^,^^10^ Some of the studies used high-risk groups, including indigenous populations, homeless individuals, or those in correctional facilities, as poverty measurements and highlighted that these groups are at high risk for the spread of resistant bacteria and the development of infection.^6^^,^^10^ However, in contrast, we used MPI to measure poverty, and findings highlighted a significant association between multi-dimensional poverty and MDR, with the likelihood of resistance increasing across poorer subgroups. Even after adjusting for key mediators like antibiotic misuse and WASH practices, poverty remained an independent predictor of MDR. This relationship can be explained by the higher burden of infections,^2^ suboptimal access to healthcare, and frequent antibiotic misuse in deprived settings.^9^ Secondly, poverty is also associated with malnutrition, which can weaken the immune system, making individuals more vulnerable to infection.^30^ Thus, it increased the use of antibiotics. Previous studies have reported that socioeconomic deprivation contributes to antibiotic resistance due to increased dependence on unprescribed antibiotics, often fueled by financial constraints and limited access to healthcare professionals,^9^ which is consistent with our findings. In such populations, antibiotics are frequently misused to manage recurring infections resulting from poor living conditions, inadequate sanitation, and limited preventive healthcare services.^3^

Poverty can play a role in shaping treatment-seeking behavior.^31^ In the current study, deprived patients showed a significant delay in infection treatment. The delayed behavior may be associated with financial constraints and limited healthcare access, as in the current study, most patients were vocal about challenges related to financial constraints in healthcare access. Others supported our findings, which focused on the importance of healthcare services' geographical location and monetary constraints in making the treatment decision.^15^ In such scenarios, the infection remains untreated or treated inadequately, which allows bacteria to persist and worsen, providing an opportunity to develop resistance mechanisms for inadequate use of antibiotics. In addition, prolonged infection may lead to self-medication with left-over antibiotics and increase the likelihood of resistance.^16^ Moreover, the extended period of infectiousness increases the risk of transmission of these resistant bacteria and exacerbates the public health challenge.

Based on data from Pakistan, the current study highlighted that multi-dimensional poverty significantly drives antibiotic resistance. However, these findings is not only constrain to Pakistan's context but also a pervasive challenge across many developing countries, where poverty, inadequate WASH conditions, delayed treatment, inability to meet the healthcare cost, poor regulatory policies, and healthcare infrastructure create a fertile ground for the spread and emergency of antibiotic resistance.^9^^,^^12^^,^^31^ Therefore, we assumed that these results could be generalizable to developing countries. However, the differences in the healthcare system and policies, culture, and infection dynamics across LMICs need further validation of the role of poverty in antibiotic resistance.

This study has some limitations. First, the sample consists of individuals using public healthcare services, which may not reflect the wider population, including those who avoid or cannot access healthcare. However, it's noteworthy that the patients attending public healthcare come from diverse socioeconomic backgrounds, with a significant proportion belonging to vulnerable socioeconomic groups. In the current study, more than 50% of the patients were at some level of deprivation. These patients experience disproportionate challenges to healthcare access and are at an increased risk of antibiotic misuse and resistance. As a result, the insight provided by this study is crucial in designing interventions for such high-risk populations. Second, our research relies on self-reported data for poverty measurement, antibiotic misuse, and WASH practices, which can be prone to recall bias. However, we tried to minimize recall bias using a structural data collection approach. Third, the results may not apply to other infections, such as respiratory infections, where antibiotic misuse and resistance patterns vary due to differences in treatment protocols, disease progress, and patient behavior. However, in the current study, the focus on UTI was intentional, as it was a reliable lens for studying the relationship between poverty and antibiotic resistance, especially in public healthcare facilities. This approach broadens the representation of the study sample for vulnerable populations. Lastly, our study relied on prevalent cases of antibiotic resistance identified through a cross-sectional design; we are unable to distinguish between factors that influence the development (incidence) of resistance and those related to the persistence or survival of resistant infections. This restricts our capacity to establish temporal or causal relationships between poverty and antibiotic resistance.

### Conclusion

The current study highlighted that poverty plays a significant role in antibiotic resistance. Poverty could shape delayed treatment-seeking and antibiotic misuse behavior, which leads to antibiotic resistance. Although the change in individual behavior is important, however, structural barriers that promote antibiotic misuse and resistance, including inaccessibility to the healthcare system, unaffordability of healthcare services, and poor living standards, should be acknowledged in designing the policies to promote rational antibiotic use and mitigate antibiotic resistance.

## Contributors

IH conceptualized the paper, analyzed quantitative data, and wrote the manuscript. YF conceptualized the paper, co-designed the study, supervised data collection, wrote the manuscript, and had the final responsibility for the decision to submit for publication. JC conceptualized the paper, co-designed the study, supervised data analysis, and wrote the manuscript. MFR coordinated the study and reviewed the manuscript. JU supervised data collection and reviewed the manuscript. MN supervised data collection and reviewed the manuscript. IK supervised data collection and reviewed the manuscript. MK provided statistical advice and edited the manuscript. MX coordinated the study and reviewed the manuscript. CT assisted with questionnaire development and reviewed the manuscript. YD assisted with data preparation and reviewed the manuscript. WZ assisted with data preparation and reviewed the manuscript.

## Data sharing statement

Data is available on request from the corresponding authors.

## Declaration of interests

The authors declare that there is no conflict of interest.
