# Supplementary material for: Exploring the association between multi-dimensional poverty and antibiotic resistance: findings from a mixed-methods study in Pakistan
Source: Lancet Reg Health Southeast Asia. 2025 Aug 27;41:100656. doi: 10.1016/j.lansea.2025.100656 (PMC12409374; doi:10.1016/j.lansea.2025.100656)
Supplement: Supplementary Figures and Tables [file mmc1.docx]

**Supplementary information**

Contents

[1. Supplementary information related to the method 2](#_Toc203728903)

[1.1 Sampling design and patients’ selection 2](#_Toc203728904)

[1.2 Assessment of Multidimensional Poverty 4](#_Toc203728905)

[1.3 Reliability analysis of the study questionnaire 5](#_Toc203728906)

[1.4 Detailed study questionnaires 5](#_Toc203728907)

[1.4.1 Patients’ treatment-seeking pathways 5](#_Toc203728908)

[1.4.2 Antibiotics misuse 6](#_Toc203728909)

[1.4.3 WASH practices 6](#_Toc203728910)

[1.5 Antibiotic susceptibility data 7](#_Toc203728911)

[1.6 Topic guides for Qualitative - in-depth interviews 9](#_Toc203728912)

[2 Supplementary information related to the results 10](#_Toc203728913)

[2.1 Distribution of the demographics across the provinces 10](#_Toc203728914)

[2.2 Distribution of antibiotic misuse across poverty dimensions 11](#_Toc203728915)

[2.3 Distribution of the WASH practices across the poverty dimensions 11](#_Toc203728916)

[2.4 Antibiotic resistance 14](#_Toc203728917)

[2.5 Role of multi-dimensional poverty in treatment-seeking behavior 16](#_Toc203728918)

[2.6 Association of the multi-dimension poverty with MDR 18](#_Toc203728919)

[Association of antibiotic misuse, WASH practices and demographic variables with MDR 33](#_Toc203728920)

[2.7 33](#_Toc203728925)

[Association of multi-dimension poverty with antibiotic misuse indicators 35](#_Toc203728926)

[2.8 35](#_Toc203728932)

[2.9 Association of multi-dimension poverty with WASH practices 35](#_Toc203728933)

# **Supplementary information related to the method**

## **Sampling design and patients’ selection**

Pakistan has four provinces (Punjab, Sindh, Khyber Pakhtunkhwa, and Balochistan) and two semi-autonomous regions (Azad Jamu Kashmir and Gilgit Baltistan). By population, Punjab is the largest province, followed by Sindh, Khyber Pakhtunkhwa, and Balochistan. Punjab has the highest literacy rate, followed by Sindh, Khyber Pakhtunkhwa, and Balochistan. Overall, Punjab and Sindh have a lower incidence of poverty, whereas Khyber Pakhtunkhwa and Balochistan experience moderate and high poverty levels, respectively. The details are given at: Pakistan Social and Living Standards Measurement Survey (PSLM) 2019-20 (<https://www.pbs.gov.pk/publication/pakistan-social-and-living-standards-measurement-survey-pslm-2019-20-provincial>) and Pakistan Economic Survey (PES) 2022-23 (<https://www.finance.gov.pk/survey_2023.html>). Thus, from the given information, we selected three provinces (Punjab, Khyber Pakhtunkhwa, and Balochistan). We excluded Sindh because it has diffusible parameters between Punjab and Khyber Pakhtunkhwa in terms of education, gross domestic product, and poverty in rural regions (**Figure S1**). All participants in the study were Pakistani citizens living in Punjab, Khyber Pakhtunkhwa, or Balochistan provinces.

The decision to choose patients from laboratories rather than hospitals was based on a pilot discussion with four urologists from three provinces (Punjab, Khyber Pakhtunkhwa, and Balochistan), and the likelihood of patients visiting physicians and recommending susceptibility testing is extremely low. That’s why we followed a path from the lab to the physician instead of from the physician to the lab (**Figure S2**).


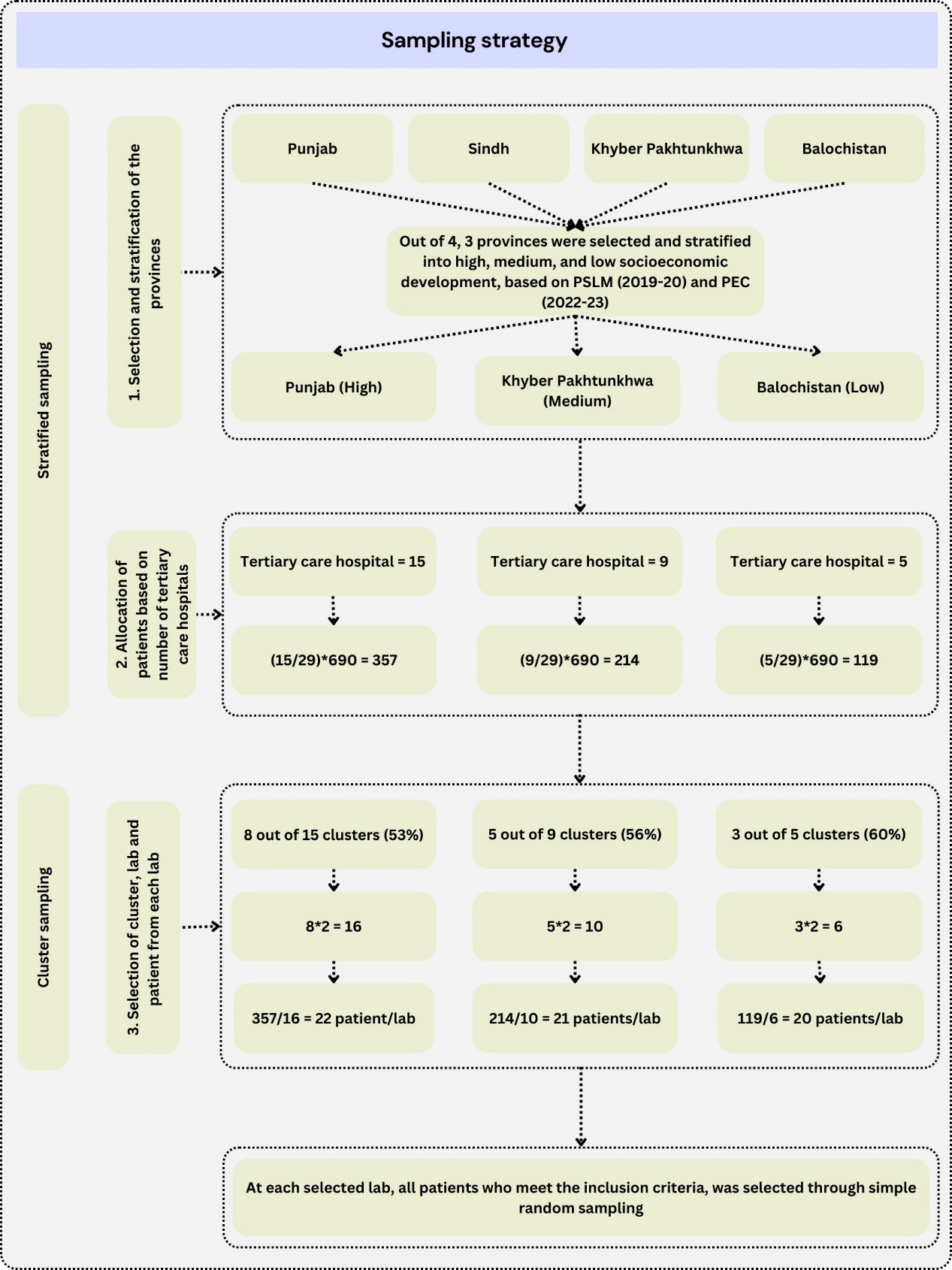


**Figure S1. Flow chart of the sampling strategy (PSLM – Pakistan Social and Living Standards Survey, PES – Pakistan Economic Survey)**


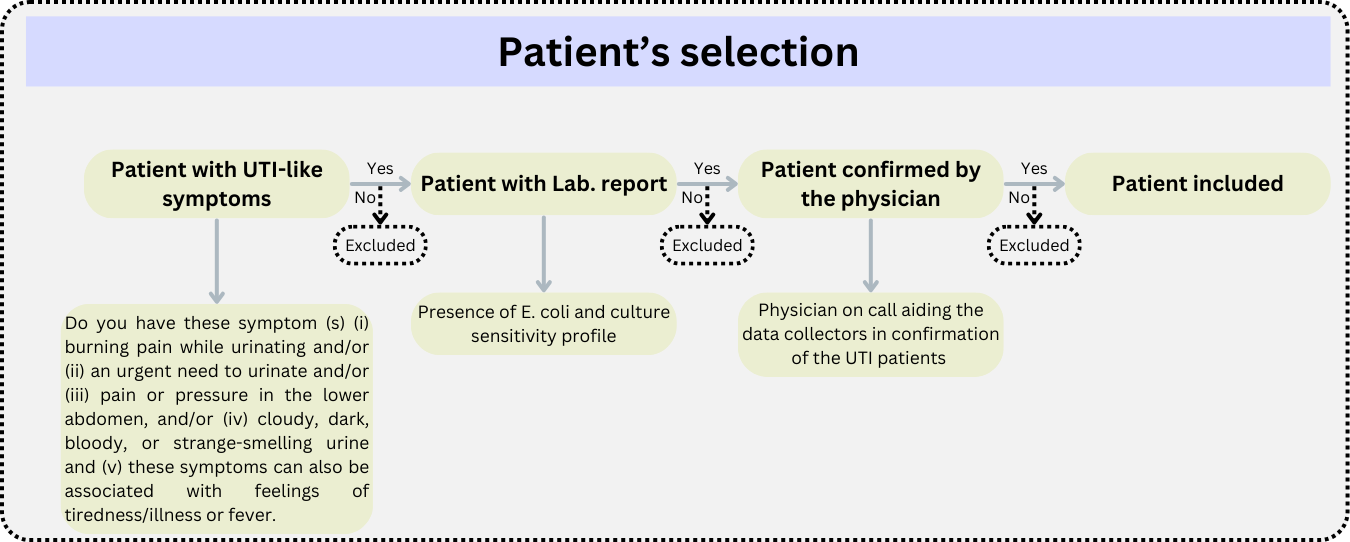


**Figure S2. Flow chart of the patient's selection (UTI – Urinary Tract Infection)**

## **Assessment of Multidimensional Poverty**

Multidimensional poverty was evaluated at the individual level with a scoring tool adapted from the HATUA (Holistic Approach to Unravel Antibacterial Resistance in East Africa) study. ^1^ The tool included seven indicators across three dimensions: education, health, and standard of living. Each indicator was evaluated as binary (Yes (deprived) = 1, No (not deprived) = 0) and weighted based on its contribution to overall deprivation.

Based on each patient’s calculated domain-weighted MPI score, we classified participants into four poverty levels, following the thresholds used in previous studies^1,2^ (**Table S1**). Patients with an MPI score below 20% were classified as not deprived. Those scoring between 20% and less than 34% were deemed vulnerable to poverty. Scores from 34% to 49% indicated deprivation, while individuals with MPI scores of 50% or higher were identified as living in severe poverty.

**Supplementary Table S 1. Dimensions, Indicators, Deprivation Cut-offs, and Weights for MPI Scoring (source: Green et al, 2023)^1^**

| **Dimension** | **Indicator** | **Deprivation Cut-off** | **Weight** |
| --- | --- | --- | --- |
| **Education** | Level of education | The patient has less than a secondary education | 1/3 |
| **Health** | Disability | Patient reports a disability | 1/6 |
|  | Chronic illness | Patients report one or more chronic illnesses (e.g., diabetes, asthma, hypertension, HIV/AIDS, stroke, cancer) | 1/6 |
| **Standard of Living** | Sanitation | No access to a private flush toilet | 1/12 |
|  | Asset ownership | Owns one or none of the following: TV, computer, radio, fridge, phone, boat, motor | 1/12 |
|  | Electricity access | No access to mains electricity | 1/12 |
|  | Drinking water source | No access to a private protected water source | 1/12 |

## **Reliability analysis of the study questionnaire**

The reliability analysis is given in **Table S1**. Overall, the questionnaire showed valid internal consistency. However, the animal ownership section showed a low reliability coefficient (0·11). This may be due to its singular nature; it contains only one primary question with follow-up questions based on a "yes" response.

**Table S1. Reliability analysis of the study questionnaire**

|  | **Average inter item correlation** | **Scale reliability coefficient** |
| --- | --- | --- |
| Patient’s pathways | 0·31 | 0·57 |
| Antibiotic misuse | 0·14 | 0·88 |
| WASH – hand washing | 0·32 | 0·65 |
| WASH – drinking water | 0·49 | 0·83 |
| WASH- Toilet/Trash | 0·34 | 0·61 |
| WASH – Animal ownership | 0·009 | 0·11 |
| WASH – Milk use | 0·03 | 0·52 |
| WASH – Overall | 0·09 | 0·90 |
| Overall | 0·03 | 0·79 |

WASH – Water sanitation and hygiene

**Note**: A standardized Cronbach alpha was used because the items were on a different scale

## **Detailed study questionnaires**

### **Patients’ treatment-seeking pathways**

The treatment-seeking pathways measure the symptoms, treatment delays, treatment-seeking behavior, and treatment used (**Table S2**).

**Table S2. Patients’ treatment-seeking pathways**

| 1. **How long did it take before you tried to treat these symptoms?** | | | |
| --- | --- | --- | --- |
| - *Immediately* | - *1-week* | - *2-weeks* | - *3-weeks* |
| - *>3weeks* |  |  |  |
| 1. **How did you seek treatment?** | | | |
| - *Govt. Hospital* | - *Private hospital* | - *Pharmacy Drug store* | - *Self-treatment* |
| 1. **What was the treatment that you tried** | | | |
| - *No medicine* | - *Antibiotics* | - *Other medicine* | |

### **Antibiotics misuse**

**Table S3. Questionnaire assessing the antibiotic misuse**

| 1. **Did you use antibiotic as a self-treatment?** | |
| --- | --- |
| - *Yes* | *No* |
| 1. **Did you complete the antibiotic course** | |
| - *Yes* | *No* |
| 1. **Did you skip the dose** | |
| - *Yes* | *No* |

### **WASH practices**

The WASH questionnaire was comprised of five sections. The first section was related to hand-washing practices. The second section assessed drinking water sources, storage and cleaning practices. The details regarding toilets and trash disposal were assessed in section 3. Sections 4 and 5 evaluate the animal living and milk use practices. The details are given in **Table S4**.

**Table S4. Questionnaire assessing the WASH practices**

| 1. **Hand washing** | | | | | | | | | | | |
| --- | --- | --- | --- | --- | --- | --- | --- | --- | --- | --- | --- |
| - 1. **Do you wash your hands with water for any reason? *** | | | | | | | | | | | |
| *Yes* | *No* | | | | | |  | | | |  |
| - 1. **Do you observe your family washing hands. *** | | | | | | | | | | | |
| *Yes* | *No* | | | | | |  | | | |  |
| - 1. **Do you use soup for hand washing? *** | | | | | | | | | | | |
| *Yes* | *No* | | | | | |  | | | |  |
| - 1. **Do you use hand sanitizer instead of soap and water? *** | | | | | | | | | | | |
| *Yes* | *No* | | | | | |  | | | |  |
| - 1. **Where did the water come from that you used to wash your hands? *** | | | | | | | | | | | |
| *Protected stored tank* | | | | | *Standing water from bucket* | | | | | | |
| 1. **Drinking water** | | | | | | | | | | | |
| - 1. **What is your primary source of drinking water?** | | | | | | | | | | | |
| *Bottled water* | *Tap water* | | | | | | *Well water* | | | | *Spring water* |
| *Rainwater* | *Other* | | | | | |  | | | |  |
| - 1. **How did you store drinking water?** | | | | | | | | | | | |
| *Water tanks covered* | *Water tanks uncovered* | | | | | | *Water container covered* | | | | *Water container uncovered* |
| - 1. **Are the storage containers/tanks cleaned before they are used?** | | | | | | | | | | | |
| *Yes* | *No* | | | | | |  | | | |  |
| - 1. **Do you treat your drinking water before using it?** | | | | | | | | | | | |
| *Yes* | *No* | | | | | |  | | | |  |
| - 1. **How often do you disinfect your drinking water containers?** | | | | | | | | | | | |
| *Never* | *Sometime* | | | | | | *Always* | | | |  |
| 1. **Toilet/latrine facility and Trash disposal** | | | | | | | | | | | |
| - 1. **Do you have toilet/latrine facility in your house?** | | | | | | | | | | | |
| *Yes* | | | *No (go to 3.5)* | | | | |  | | |  |
| - 1. **What kind of toilet facility or latrine do members of your household use most often?** | | | | | | | | | | | |
| *Open defecation* | *Pit latrine* | | | | | | *Flush toilet* | | | | *Others* |
| - 1. **Where is the waste flushed?** | | | | | | | | | | | |
| *Flushed to piped sewer system* | | | | *Flushed to pit latrine* | | | | | *No Drainage/overflows to the floor* | | |
| - 1. **Do you share this toilet/latrine or defecation field with other households?** | | | | | | | | | | | |
| *Yes* | *No* | | | | | |  | | | |  |
| - 1. **How do you dispose of the trash in your household?** | | | | | | | | | | | |
| *Open burning* | *Burred as fuel at home* | | | | | | *Designated pit* | | | | *Don't know* |
| 1. **Household animals** | | | | | | | | | | | |
| - 1. **Do you own domestic animals?** | | | | | | | | | | | |
| *Yes* | *No (go to 5)* | | | | | | | | | |  |
| - 1. **If yes, then state the name and quantity of the animal: ____________________________** | | | | | | | | | | | |
| - 1. **Have you ever given antibiotics to your animals?** | | | | | | | | | | | |
| *Yes* | *No* | | | | | |  | | | |  |
| - 1. **Do you have a domestic animal that lives freely inside the house?** | | | | | | | | | | | |
| *Yes* | *No* | | | | | |  | | | |  |
| - 1. **Are your animals sometimes kept inside the living quarters (in the day or the night)?** | | | | | | | | | | | |
| *Yes* | *No* | | | | | |  | | | |  |
| 1. **Milke use** | | | | | | | | | | | |
| - 1. **Do you use milk?** | | | | | | | | | | | |
| *Yes* | | *No (go to submission)* | | | | | | | |  |  |
| - 1. **Do you boil the milk before taking it?** | | | | | | | | | | | |
| *Yes* | *No* | | | | | |  | | | |  |
| - 1. **5.14.2 Do you store Milk?** | | | | | | | | | | | |
| *Yes* | | | | | | *No (go to 5.5)* | | | | | |
| - 1. **Where you store milk** | | | | | | | | | | | |
| *Open space* | *Refrigerator* | | | | | |  | | | |  |
| - 1. **Do you wash the container before putting milk in it?** | | | | | | | | | | | |
| *Yes* | *No* | | | | | |  | | | |  |

## **Antibiotic susceptibility data**

In Pakistan, antibiotic prescribing practices for urinary tract infections (UTIs) often reflect patterns of resistance and the availability of medications ^3^. Secondly, guidelines exist for antibiotic prescription in UTI, but adherence varies among healthcare providers in different provinces ^4,5^. To address this issue, we used a standardized pre-developed tool for antibiotic inclusion and resistance categorization for UTI (**Table S5**). In addition, we used the WHO AWaRe (Access, Watch, Reserve) classification to deepen the situation of antibiotic resistance.

**Table S5. Antimicrobial categories and agents to present antibiotic resistance**

| **Antimicrobial category** | **Antimicrobial agents** | **AWaRe Category** | **ATC code** |
| --- | --- | --- | --- |
| Aminoglycosides | Gentamicin | Access | J01GB03 |
|  | Amikacin | Access | J01GB06 |
|  | Tobramycin | Watch | J01GB01 |
|  | Netilmicine | Watch | J01GB07 |
| Anti-MRSA cephalosporines | Ceftaroline | Reserve | J01DI02 |
| Antipseudomonal penicillins + β-lactamase inhibitors | Ticarcillin-clavulanic acid | Watch | J01CA13 |
|  | Piperacillin-tazobactam | Watch | J01CR05 |
| Carbapenems | Imipenem | Watch | J01DH51 |
|  | Meropenem | Watch | J01DH02 |
|  | Doripenem | Watch | J01DH04 |
|  | Ertapenem | Watch | J01DH03 |
| Non-extended spectrum cephalosporins; 1st and 2nd generation cephalosporins | Cefazolin | Access | J01DB04 |
|  | Cefuroxime | Watch | J01DC02 |
| Extended-spectrum cephalosporins; 3rd and 4th generation cephalosporins | Cefotaxime | Watch | J01DD01 |
|  | ceftriaxone | Watch | J01DD04 |
|  | Ceftazidime | Watch | J01DD02 |
|  | Cefepime | Watch | J01DE01 |
| Cephamycins | Cefoxitin | Watch | J01DC01 |
|  | Cefotetan | Watch | J01DC05 |
| Fluoroquinolones | Ciprofloxacin | Watch | J01MA02 |
| Folate pathway inhibitors | Trimethoprim-sulphamethoxazole | Access | J01EE01 |
| Glycylcyclines | Tigecycline | Reserve | J01AA12 |
| Monobactams | Aztreonam | Reserve | J01DF01 |
| Penicillins | Ampicillin | Access | J01CA01 |
| Penicillins + β-lactamase inhibitors | Amoxicillin-clavulanic acid | Access | J01CR02 |
|  | Ampicillin-sulbactam | Access | J01CR01 |
| Phenicols | Chloramphenicol | Access | J01BA01 |
| Phosphonic acids | Fosfomycin | Watch | J01XX01 |
| Polymyxins | Colistin | Reserve | A07AA10 |
| Tetracyclines | Tetracycline | Access | J01AA07 |
|  | Doxycycline | Access | J01AA02 |
|  | Minocycline | Watch | J01AA08 |
| **Multidrug-resistant (MDR)**: non-susceptible to ≥1 agent in >3 antimicrobial categories. **Extensively drug-resistant** (**XDR)**: non-susceptible to ≥1 agent in all but ≤2 categories. **Pandrug-resistant (PDR)**: non-susceptible to all antimicrobial agents listed. | | | |

##

## **Topic guides for Qualitative - in-depth interviews**

**Table S6. Topic guides for Qualitative part - in-depth interviews**

| Patient ID code (same as quantitative part) |  |
| --- | --- |
| Study area name |  |
| Date |  |
| Time interview started and ended |  |
| Interviewers name |  |
| Was any other person present during the interview – please explain |  |
| **Instruction to interviewers** | |
| 1. Use simple, clear language and avoid medical jargon. 2. Start with general, non-threatening questions to make participants comfortable. 3. Show empathy and respect for their experiences. 4. Follow up on initial responses with probing questions to gather more detailed information.   For example, "Can you tell me more about that?" or "Why do you think that happens?"   1. Be aware of cultural norms and practices. 2. Respect the participants' beliefs and values during the interview. 3. Conduct interviews in a private and comfortable setting to encourage openness. 4. Assure participants that their responses will be confidential. | |
| **Questions** | |
| 1. What do you know about antibiotics and antibiotics resistance? (knowledge about antibiotics, antibiotic resistance, source of information) 2. Can you tell me about the nearest healthcare facility to your home? How often do you visit it? 3. What challenges do you face in accessing healthcare services? 4. When you or your family members get UTI, where do you usually get medicines from? 5. Have you ever had difficulties in getting prescribed medicines, especially for UTI? If so, what were the reasons? 6. Have you ever used antibiotics without a prescription, especially for UTI? If yes, can you share why and how often this happens? 7. Do you complete the full course of antibiotics when prescribed for UTI? 8. How much of your income is typically spent on healthcare and medicines? 9. Have you ever had to choose between buying food and buying medicines? Can you share an example? 10. Can you describe your living conditions? (e.g., type of housing, number of people living in the house) 11. How would you rate the cleanliness and sanitation in your home? 12. What measures do you take to maintain hygiene at home? (e.g., handwashing, clean water) 13. Do you have access to clean drinking water and proper sanitation facilities? 14. Are there any traditional beliefs or practices related to UTI treatment and medication in your community? 15. How do these beliefs influence your decisions about using antibiotics? | |

# **Supplementary information related to the results**

## **Distribution of the demographics across the provinces**

**Table S7** shows the demographic distribution across the provinces. Nuclear families are more prevalent in Punjab, whereas joint/extended families are relatively higher in Balochistan. Employment rates are higher in Punjab (54·5%) compared to KPK (35·1%) and Balochistan (10·4%).

**Table S7. Distribution of the demographics across the provinces**

|  | **Province** | | |
| --- | --- | --- | --- |
|  | **Punjab** | **Kpk** | **Balochistan** |
|  | **N (%)** | **N (%)** | **N (%)** |
| **Gender** | | | |
| Male | 159 (52·8) | 85 (28·2) | 57 (18·9) |
| Female | 198 (49·9) | 136 (34·3) | 63 (15·9) |
| **Age** | | | |
| 18-30 | 97 (44·7) | 85 (39·2) | 35 (16·1) |
| 31-40 | 97 (57·7) | 46 (27·4) | 25 (14·9) |
| 41-50 | 87 (59·6) | 31 (21·2) | 28 (19·2) |
| 51-60 | 39 (47·6) | 24 (29·3) | 19 (23·2) |
| >60 | 37 (43·5) | 35 (41·2) | 13 (15·3) |
| **Marital status** | | | |
| Single | 72 (50·0) | 48 (33·3) | 24 (16·7) |
| Married | 274 (51·9) | 163 (30·9) | 91 (17·2) |
| Other | 11 (42·3) | 10 (38·5) | 5 (19·2) |
| **Family structure** | | | |
| Nuclear family | 148 (55·4) | 83 (31·1) | 36 (13·5) |
| Joint/extended family | 209 (48·5) | 138 (32·0) | 84 (19·5) |
| **Number of family member** | | | |
| 1-2 | 61 (51·3) | 45 (37·8) | 13 (10·9) |
| 3-6 | 109 (51·9) | 65 (31·0) | 36 (17·1) |
| >6 | 187 (50·7) | 111 (30·1) | 71 (19·2) |
| **Education** | | | |
| No education | 114 (51·4) | 60 (27·0) | 48 (21·6) |
| Primary | 80 (46·5) | 57 (33·1) | 35 (20·3) |
| Secondary | 87 (61·3) | 40 (28·2) | 15 (10·6) |
| >Secondary | 76 (46·9) | 64 (39·5) | 22 (13·6) |
| **Employment** |  |  |  |
| Not employed | 236 (49·6) | 143 (30·0) | 97 (20·4) |
| Employed | 121 (54·5) | 78 (35·1) | 23 (10·4) |
| **Ability to meet healthcare cost** | | | |
| Easy | 85 (47·8) | 73 (41·0) | 20 (11·2) |
| Little difficult | 160 (53·2) | 96 (31·9) | 45 (15·0) |
| Very difficult | 112 (51·1) | 52 (23·7) | 55 (25·1) |
| **UTI history** | | | |
| No | 148 (48·5) | 93 (30·5) | 64 (21·0) |
| Yes | 209 (53·2) | 128 (32·6) | 56 (14·2) |
| **Symptom stigma** | | | |
| No | 231 (52·9) | 115 (26·3) | 91 (20·8) |
| Yes | 126 (48·3) | 106 (40·6) | 29 (11·1) |
| **Source of healthcare advice** | | | |
| Fromal | 177 (48·0) | 136 (36·9) | 56 (15·2) |
| Informal | 180 (54·7) | 85 (25·8) | 64 (19·5) |

## **Distribution of antibiotic misuse across poverty dimensions**

The prevalence of self-medication increases significantly with the level of deprivation, being highest among the severely deprived group (42·3%). The severely deprived group shows the highest incompletion rates (50·7%). The details are given in **Table S8**.

**Table S8. Distribution of antibiotic misuse across the poverty dimension**

|  | **Multi-dimensional poverty** | | | |
| --- | --- | --- | --- | --- |
|  | **Not deprived** | **Vulnerable** | **Deprived** | **Severely deprived** |
|  | **N (%)** | **N (%)** | **N (%)** | **N (%)** |
| Self-medication | | | | |
| No | 159 (68·5) | 30 (12·9) | 20 (8·6) | 23 (9·9) |
| Yes | 126 (27·0) | 87 (18·7) | 56 (12·0) | 197 (42·3) |
| Antibiotic course completion | | | | |
| No | 85 (22·9) | 52 (14·0) | 46 (12·4) | 188 (50·7) |
| Yes | 200 (61·2) | 65 (19·9) | 30 (9·2) | 32 (9·8) |
| Dose skipping | | | | |
| No | 189 (63·4) | 53 (17·8) | 28 (9·4) | 28 (9·4) |
| Yes | 96 (24·0) | 64 (16·0) | 48 (12·0) | 192 (48·0) |

## **Distribution of the WASH practices across the poverty dimensions**

The prevalence of not washing hands is highest in the severely deprived group (53·5%), relying on unprotected water sources (60·9%). Most participants across poverty dimensions use well water; however, in the severely deprived level, individuals predominantly use unsafe sources like rainwater (100%). The details are given in **Table S9.**

**Table S9. Distribution of the WASH practices across the poverty dimensions**

|  | **Multi-dimensional poverty** | | | |
| --- | --- | --- | --- | --- |
|  | **Not deprived** | **Vulnerable** | **Deprived** | **Severely deprived** |
|  | **N (%)** | **N (%)** | **N (%)** | **N (%)** |
| **WASH Practices** | | | | |
| **Hand washing** | | | | |
| Washing hands – self | | | | |
| No | 12 (14·0) | 17 (19·8) | 11 (12·8) | 46 (53·5) |
| Yes | 273 (44·6) | 100 (16·3) | 65 (10·6) | 174 (28·4) |
| Washing hands – family | | | | |
| No | 140 (32·3) | 81 (18·7) | 59 (13·6) | 153 (35·3) |
| Yes | 145 (54·7) | 36 (13·6) | 17 (6·4) | 67 (25·3) |
| Use soup for hand washing | | | | |
| No | 218 (36·2) | 99 (16·4) | 73 (12·1) | 213 (35·3) |
| Yes | 67 (70·5) | 18 (18·9) | 3 (3·2) | 7 (7·4) |
| Use hand sanitizer if soap and water not available | | | | |
| No | 281 (41·0) | 116 (16·9) | 73 (10·6) | 216 (31·5) |
| Yes | 4 (33·3) | 1 (8·3) | 3 (25·0) | 4 (33·3) |
| Hand washing water source | | | | |
| Unprotected | 41 (13·1) | 35 (11·2) | 46 (14·7) | 190 (60·9) |
| Protected | 244 (63·2) | 82 (21·2) | 30 (7·8) | 30 (7·8) |
| **Drinking water** | | | | |
| Drinking water source | | | | |
| Rainwater | 0 (0·0) | 0 (0·0) | 0 (0·0) | 36 (100·0) |
| Spring water | 0 (0·0) | 0 (0·0) | 1 (4·8) | 20 (95·2) |
| Well water | 270 (44·8) | 104 (17·2) | 71 (11·8) | 158 (26·2) |
| Tap water | 13 (50·0) | 4 (15·4) | 4 (15·4) | 5 (19·2) |
| Bottled water | 2 (16·7) | 9 (75·0) | 0 (0·0) | 1 (8·3) |
| Drinking water storage | | | | |
| Tank/container uncovered | 15 (13·8) | 6 (5·5) | 10 (9·2) | 78 (71·6) |
| Tank/container covered | 270 (45·8) | 111 (18·8) | 66 (11·2) | 142 (24·1) |
| Storage containers/tanks cleaned before they are used | | | | |
| No | 146 (30·0) | 83 (17·0) | 68 (14·0) | 190 (39·0) |
| Yes | 139 (65·9) | 34 (16·1) | 8 (3·8) | 30 (14·2) |
| Drinking water treatment before use | | | | |
| No | 20 (8·6) | 10 (4·3) | 37 (15·9) | 165 (71·1) |
| Yes | 265 (57·0) | 106 (22·8) | 39 (8·4) | 55 (11·8) |
| How often do you disinfect your drinking water containers | | | | |
| Never | 106 (24·3) | 65 (14·9) | 61 (14·0) | 204 (46·8) |
| Sometime | 104 (59·8) | 43 (24·7) | 13 (7·5) | 14 (8·0) |
| Often | 75 (85·2) | 9 (10·2) | 2 (2·3) | 2 (2·3) |
| **Toilet/Latrine Facility** | | | | |
| Availability of toilet/latrine facility in the house | | | | |
| No | 9 (4·9) | 6 (3·2) | 35 (18·9) | 135 (73·0) |
| Yes* | 276 (53·8) | 111 (21·6) | 41 (8·0) | 85 (16·6) |
| Type of toilet/latrine facility availability in the house | | | | |
| Open defecation | 9 (4·9) | 6 (3·2) | 35 (18·9) | 135 (73·0) |
| Pit latrine | 23 (21·7) | 14 (13·2) | 9 (8·5) | 60 (56·6) |
| Flush toilet | 253 (62·2) | 97 (23·8) | 32 (7·9) | 25 (6·1) |
| Toilet/latrine waste flushed into | | | | |
| No drainage | 0 (0·0) | 4 (80·0) | 0 (0·0) | 1 (20·0) |
| Pit latrine | 23 (21·7) | 14 (13·2) | 9 (8·5) | 60 (56·6) |
| Piped sewer system | 253 (62·9) | 93 (23·1) | 32 (8·0) | 24 (6·0) |
| Sharing toilet/latrine facility with other family member | | | | |
| No | 222 (56·1) | 86 (21·7) | 34 (8·6) | 54 (13·6) |
| Yes | 63 (20·9) | 31 (10·3) | 42 (13·9) | 166 (55·0) |
| **Dispose of the trash** | | | | |
| How do you dispose of the trash in your household | | | | |
| Burned as fuel at home | 100 (40·5) | 30 (12·1) | 27 (10·9) | 90 (36·4) |
| Open burning | 33 (41·8) | 16 (20·3) | 3 (3·8) | 27 (34·2) |
| Designated pit | 152 (40·9) | 71 (19·1) | 46 (12·4) | 103 (27·7) |
| **Domestic animals** | | | | |
| Own domestic animals | | | | |
| No | 110 (43·3) | 34 (13·4) | 29 (11·4) | 81 (31·9) |
| Yes* | 175 (39·4) | 83 (18·7) | 47 (10·6) | 139 (31·3) |
| Use of antibiotics for domestic animal | | | | |
| No | 82 (44·3) | 30 (16·2) | 22 (11·9) | 51 (27·6) |
| Yes | 28 (41·8) | 4 (6·0) | 7 (10·4) | 28 (41·8) |
| Domestic animal lives freely inside the house | | | | |
| No | 86 (42·8) | 23 (11·4) | 24 (11·9) | 68 (33·8) |
| Yes | 24 (47·1) | 11 (21·6) | 5 (9·8) | 11 (21·6) |
| Domestic animals sometimes kept inside the living quarters | | | | |
| No | 98 (42·6) | 31 (13·5) | 28 (12·2) | 73 (31·7) |
| Yes | 12 (54·5) | 3 (13·6) | 1 (4·5) | 6 (27·3) |
| Do you use milk? | | | | |
| No | 53 (36·1) | 29 (19·7) | 14 (9·5) | 51 (34·7) |
| Yes* | 232 (42·1) | 88 (16·0) | 62 (11·3) | 169 (30·7) |
| Do you boil the milk before taking it? | | | | |
| No | 12 (20·3) | 4 (6·8) | 5 (8·5) | 38 (64·4) |
| Yes | 220 (44·7) | 84 (17·1) | 57 (11·6) | 131 (26·6) |
| Do you store Milk? | | | | |
| No | 8 (47·1) | 1 ()5·9 | 2 (11·8) | 6 (35·3) |
| Yes | 224 (41·9) | 87 (16·3) | 60 (11·2) | 163 (30·5) |
| Where do you store the milk? | | | | |
| Open space | 27 (47·4) | 5 (8·8) | 6 (10·5) | 19 (33·3) |
| Refrigerator | 204 (41·9) | 82 (16·8) | 55 (11·3) | 146 (30·0) |
| Do you wash the container before putting milk in it? | | | | |
| No | 11 (37·9) | 3 (10·3) | 3 (10·3) | 12 (41·4) |
| Yes | 220 (42·7) | 84 (16·3) | 58 (11·3) | 153 (29·7) |

* - represent that the subsequent questions are based on “yes” response, WASH – Water, Sanitation and Hygiene

## **Antibiotic resistance**

Multidrug-resistant (MDR) pathogens were detected in samples from more than three-quarters of the participants (77%). In the access group, Amikacin, amoxicillin-clavulanic acid, and gentamycin showed high resistance, whereas in the watch group, cefepime, ceftriaxone, ciprofloxacin, and the reserve group, aztreonam showed a high resistant profile, as shown in **Figure S3**. The distribution of the susceptibility profile across patients and poverty dimensions are given in supplementary Figures **S4** and **S5**, respectively. The distribution of sensitivity profile of antibiotics and distribution across provinces are given in **Figure S6** and **Figure S7**.


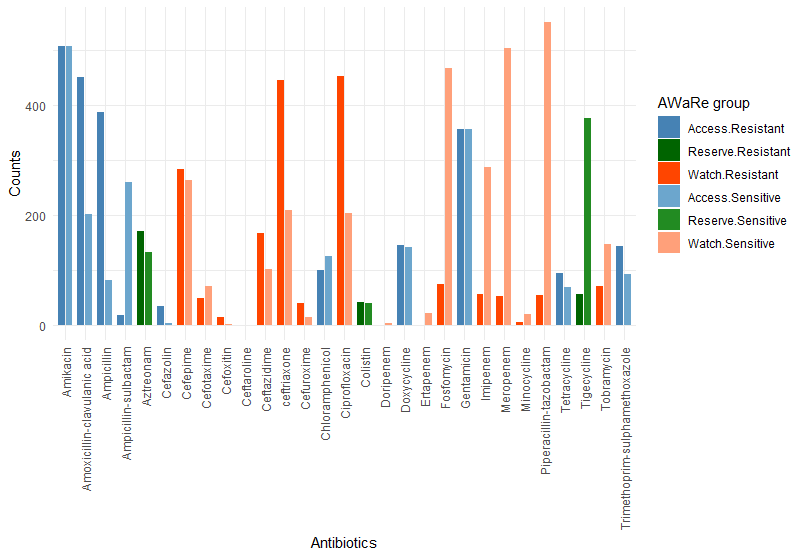


**Figure S3. Distribution of antibiotic susceptibility by AWaRe classification**

**Figure S4. Distribution of the sensitive and resistant data of included antibiotics across the patients**


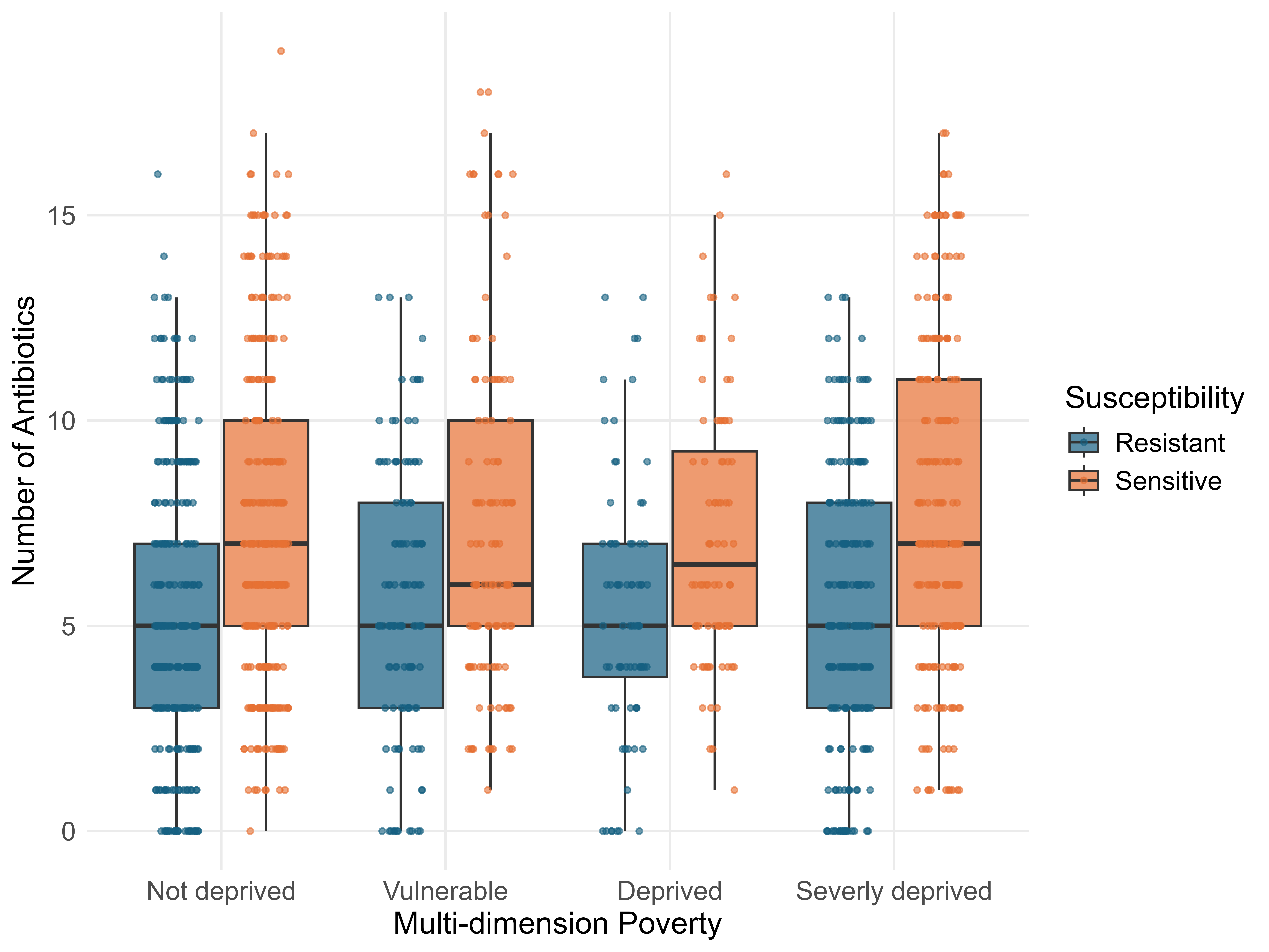


**Figure S5. Comparison of antibiotic susceptibility across the poverty dimension**





**Supplementary Figure S6. Distribution of resistant and sensitive antibiotics across three provinces in Pakistan (Punjab, Sindh, and Khyber Pakhtunkhwa (KPK))**





**Supplementary Figure S7. Distribution of resistant and sensitive antibiotics across Escherichia coli (E. coli) and Klebsiella spp.)**

## **Role of multi-dimensional poverty in treatment-seeking behavior**

Increasing the deprivation level affects treatment-seeking behavior, as shown in Figure S6. Poorer subgroups were presented with delayed treatment seeking. Those who delayed treatment >3 weeks were associated with self-treatment. Most of those who visited the government hospital, pharmacy/drug store, and self-treatment used antibiotics for UTI treatment.


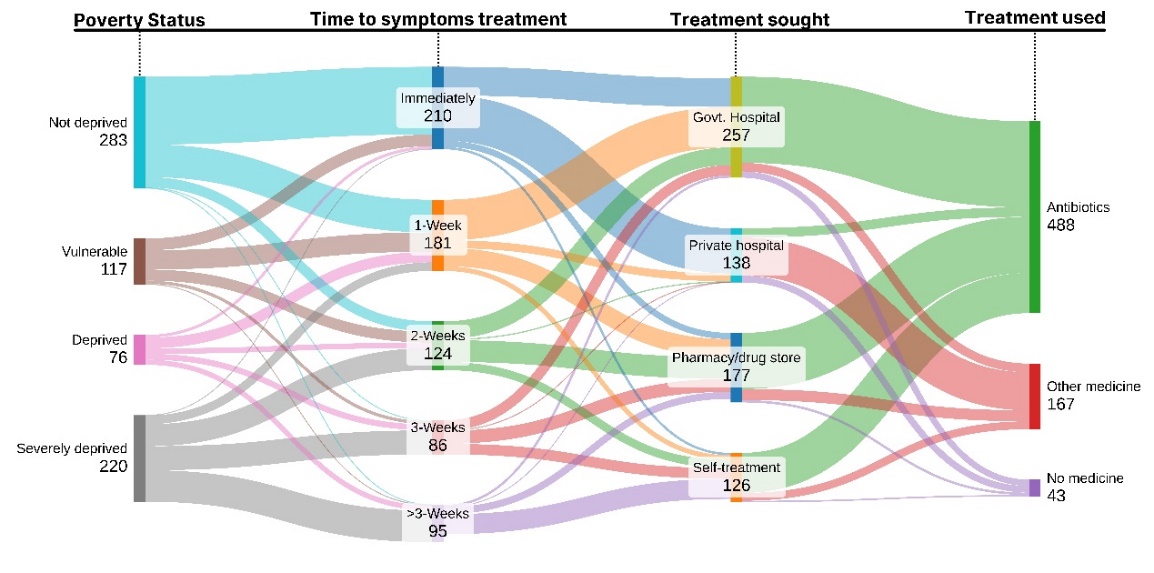


**Figure S8. Role of multi-dimensional poverty in treatment-seeking behavior**

## **Association of the multi-dimension poverty with MDR**

**Table 10 . Association of the multi-dimension poverty with multi-drug resistance (unadjusted)**

|  | **MDR** | | |
| --- | --- | --- | --- |
| **Multi-dimension Poverty** | OR (95% CI) | robust SE | P-value |
| Not deprived (Ref.) | - | - | - |
| Vulnerable | 1.94 (1.11 – 3.39) | 0.52 | 0.02 |
| Deprived | 2.05 (1.06 – 3.98) | 0.66 | 0.03 |
| Severely deprived | 1.80 (1.04 – 3.09) | 0.047 | 0.03 |

OR – Odds ratio (survey adjusted), SE – Standard error. CI – Confidence interval, Ref. – reference

**Table 11. Association of multi-dimension poverty with MDR (Model 1: adjusted for self-medication)**

| **Multi-dimension Poverty** | **aOR (95% CI)** | **robust SE** | **P-value** |
| --- | --- | --- | --- |
| Not deprived (Ref.) |  |  |  |
| Vulnerable | 1.95 (1.11 – 3.43) | 0.53 | 0.02 |
| Deprived | 2.09 (1.09 – 4.01) | 0.66 | 0.02 |
| Severely deprived | 1.83 (1.07 – 3.13) | 0.48 | 0.02 |
| Self-medication | 0.98 (0.67 – 1.43) | 0.18 | 0.93 |

OR – Odds ratio (survey adjusted), SE – Standard error. CI – Confidence interval, Ref. – reference

**Table 12. Association of multi-dimension poverty with MDR (Model 2: adjusted for course completion)**

| **Multi-dimension Poverty** | **aOR (95% CI)** | **robust SE** | **P-value** |
| --- | --- | --- | --- |
| Not deprived (Ref.) |  |  |  |
| Vulnerable | 1.91 (1.09 – 3.36) | 0.25 | 0.02 |
| Deprived | 2.02 (1.03 – 3.92) | 0.65 | 0.03 |
| Severely deprived | 1.71 (1.03 – 2.83) | 0.41 | 0.03 |
| Course completion | 0.89 (0.60 – 1.33) | 0.17 | 0.58 |

OR – Odds ratio (survey adjusted), SE – Standard error. CI – Confidence interval, Ref. – reference

**Table 13. Association of multi-dimension poverty with MDR (Model 3: adjusted for dose skipping)**

| **Multi-dimension Poverty** | **aOR (95% CI)** | **robust SE** | **P-value** |
| --- | --- | --- | --- |
| Not deprived (Ref.) |  |  |  |
| Vulnerable | 1.94 (1.11 – 3.37) | 0.52 | 0.02 |
| Deprived | 2.08 (1.08 – 3.99) | 0.66 | 0.02 |
| Severely deprived | 1.81 (1.10 – 2.97) | 0.43 | 0.02 |
| Dose skipping | 1.06 (0.69 – 1.45) | 0.18 | 0.97 |

OR – Odds ratio (survey adjusted), SE – Standard error. CI – Confidence interval, Ref. – reference

**Table 14. Association of multi-dimension poverty with MDR (Model 4: adjusted for antibiotic all misuse indicators)**

| **Multi-dimension Poverty** | **aOR (95% CI)** | **robust SE** | **P-value** |
| --- | --- | --- | --- |
| Not deprived (Ref.) |  |  |  |
| Vulnerable | 2.01 (1.13 – 3.54) | 0.55 | 0.01 |
| Deprived | 2.05 (1.06 – 3.94) | 0.65 | 0.03 |
| Severely deprived | 1.75 (1.05 – 2.91) | 0.43 | 0.03 |
| Self-medication | 0.92 (0.52 – 1.61) | 0.25 | 0.78 |
| Course completion | 0.64 (0.27 – 1.49) | 0.26 | 0.29 |
| Dose skipping | 0.72 (0.31 – 1.64) | 0.29 | 0.42 |

OR – Odds ratio (survey adjusted), SE – Standard error. CI – Confidence interval, Ref. – reference

**Table 15. Association of multi-dimension poverty with MDR (Model 5: adjusted for WASH practices)**

| **Multi-dimension Poverty** | **aOR (95% CI)** | **robust SE** | **P-value** |
| --- | --- | --- | --- |
| Not deprived (Ref.) |  |  |  |
| Vulnerable | 2.02 (1.17 – 3.50) | 0.54 | 0.01 |
| Deprived | 2.39 (1.17 – 4.89) | 0.83 | 0.01 |
| Severely deprived | 2.28 (1.19 – 4.38) | 0.72 | 0.01 |
| WASH practices | 1.37 (0.81 – 2.33) | 0.35 | 0.22 |

OR – Odds ratio (survey adjusted), SE – Standard error. CI – Confidence interval, Ref. – reference

**Table 16. Association of multi-dimension poverty with MDR (Model 6: Model 4 + Model 5)**

| **Multi-dimension Poverty** | **aOR (95% CI)** | **robust SE** | **P-value** |
| --- | --- | --- | --- |
| Not deprived (Ref.) |  |  |  |
| Vulnerable | 2.08 (1.18 – 3.68) | 0.57 | 0.01 |
| Deprived | 2.36 (1.16 – 4.78) | 0.81 | 0.01 |
| Severely deprived | 2.21 (1.16 – 4.22) | 0.69 | 0.01 |
| Self-medication | 0.93 (0.53 – 1.64) | 0.81 | 0.82 |
| Course completion | 0.59 (0.25 – 1.38) | 0.24 | 0.22 |
| Dose skipping | 0.69 (0.30 – 1.59) | 0.28 | 0.37 |
| WASH practices | 1.43 (0.85 – 2.40) | 0.36 | 0.16 |

OR – Odds ratio (survey adjusted), SE – Standard error. CI – Confidence interval, Ref. – reference

**Table 17. Association of multi-dimension poverty with MDR (Model 7: Model 6 + gender)**

| **Multi-dimension Poverty** | **aOR (95% CI)** | **robust SE** | **P-value** |
| --- | --- | --- | --- |
| Not deprived (Ref.) |  |  |  |
| Vulnerable | 2.20 (1.23 – 3.93) | 0.62 | 0.009 |
| Deprived | 2.42 (1.16 – 5.03) | 0.86 | 0.01 |
| Severely deprived | 2.30 (1.17 – 4.49) | 0.75 | 0.01 |
| Self-medication | 0.94 (0.55 – 1.63) | 0.25 | 0.84 |
| Course completion | 0.59 (0.25 – 1.38) | 0.24 | 0.22 |
| Dose skipping | 0.68 (0.29 – 1.55) | 0.27 | 0.34 |
| WASH practices | 1.45 (0.85 – 2.47) | 0.37 | 0.15 |
| Gender |  |  |  |
| Male (Ref.) |  |  |  |
| Female | 0.84 (0.59 – 1.18) | 0.14 | 0.31 |

OR – Odds ratio (survey adjusted), SE – Standard error. CI – Confidence interval, Ref. – reference

**Table 18. Association of multi-dimension poverty with MDR (Model 8: model 7 + age)**

| **Multi-dimension Poverty** | **aOR (95% CI)** | **robust SE** | **P-value** |
| --- | --- | --- | --- |
| Not deprived (Ref.) |  |  |  |
| Vulnerable | 2.28 (1.25 – 4.16) | 0.61 | 0.009 |
| Deprived | 2.52 (1.22 – 5.16) | 0.88 | 0.01 |
| Severely deprived | 3.42 (1.83 – 6.39) | 1.04 | <0.001 |
| Self-medication | 0.98 (0.55 – 1.72) | 0.26 | 0.94 |
| Course completion | 0.60 (0.26 – 1.37) | 0.24 | 0.22 |
| Dose skipping | 0.75 (0.34 – 1.67) | 0.29 | 0.48 |
| WASH practices | 1.48 (0.88 – 2.49) | 0.37 | 0.12 |
| Gender |  |  |  |
| Male (ref.) |  |  |  |
| Female | 0.79 (0.55 – 1.14) | 0.14 | 0.20 |
| Age |  |  |  |
| 18 – 30 (Ref.) |  |  |  |
| 31 – 40 | 0.81 (0.52 – 1.26) | 0.17 | 0.34 |
| 41 – 50 | 0.78 (0.41 – 1.45) | 0.23 | 0.42 |
| 51 – 60 | 0.43 (0.24 – 0.76) | 0.12 | 0.005 |
| >60 | 0.41 (0.22 – 0.77) | 0.12 | 0.007 |

OR – Odds ratio (survey adjusted), SE – Standard error. CI – Confidence interval, Ref. – reference

**Table 19. Association of multi-dimension poverty with MDR (Model 9: Model 8 + marital status)**

| **Multi-dimension Poverty** | **aOR (95% CI)** | **robust SE** | **P-value** |
| --- | --- | --- | --- |
| Not deprived (Ref.) |  |  |  |
| Vulnerable | 2.30 (1.26 – 4.21) | 0.67 | 0.008 |
| Deprived | 2.46 (1.19 – 5.07) | 0.87 | 0.01 |
| Severely deprived | 3.36 (1.82 – 6.19) | 1.01 | <0.001 |
| Self-medication | 0.99 (0.56 – 1.73) | 0.27 | 0.98 |
| Course completion | 0.59 (0.26 – 1.34) | 0.23 | 0.20 |
| Dose skipping | 0.75 (0.33 – 1.67) | 0.29 | 0.47 |
| WASH practices | 1.43 (0.85 – 2.39) | 0.35 | 0.16 |
| Gender |  |  |  |
| Male (ref.) |  |  |  |
| Female | 0.79 (0.55 – 1.14) | 0.14 | 0.21 |
| Age |  |  |  |
| 18 – 30 (Ref.) |  |  |  |
| 31 – 40 | 0.88 (0.43 – 1.83) | 0.31 | 0.73 |
| 41 – 50 | 0.85 (0.37 – 1.93) | 0.34 | 0.69 |
| 51 – 60 | 0.48 (0.22 – 1.02) | 0.17 | 0.05 |
| >60 | 0.55 (0.24 – 1.26) | 0.22 | 0.15 |
| Marital status |  |  |  |
| Unmarried (Ref.) |  |  |  |
| Married | 0.86 (0.36 – 2.04) | 0.36 | 0.72 |
| Other | 0.42 (0.12 – 1.49) | 0.26 | 0.17 |

OR – Odds ratio (survey adjusted), SE – Standard error. CI – Confidence interval, Ref. – reference

**Table 20. Association of multi-dimension poverty with MDR (Model 10: Model 9 + number of family members)**

| **Multi-dimension Poverty** | **aOR (95% CI)** | **robust SE** | **P-value** |
| --- | --- | --- | --- |
| Not deprived (Ref.) |  |  |  |
| Vulnerable | 2.17 (1.19 – 3.94) | 0.63 | 0.01 |
| Deprived | 2.19 (1.08 – 4.45) | 0.75 | 0.03 |
| Severely deprived | 3.23 (1.73 – 6.02) | 0.98 | 0.001 |
| Self-medication | 0.84 (0.47 – 1.49) | 0.23 | 0.55 |
| Course completion | 0.61 (0.26 – 1.41) | 0.25 | 0.23 |
| Dose skipping | 0.78 (0.35 – 1.75) | 0.30 | 0.54 |
| WASH practices | 1.47 (0.88 -2.46) | 0.37 | 0.13 |
| Gender |  |  |  |
| Male (ref.) |  |  |  |
| Female | 0.81 (0.58 – 1.14) | 0.13 | 0.23 |
| Age |  |  |  |
| 18 – 30 (Ref.) |  |  |  |
| 31 – 40 | 0.90 (0.44 – 1.82) | 0.31 | 0.76 |
| 41 – 50 | 0.84 (0.36 – 1.95) | 0.36 | 0.68 |
| 51 – 60 | 0.48 (0.22 – 1.03) | 0.17 | 0.05 |
| >60 | 0.54 (0.23 – 1.29) | 0.22 | 0.16 |
| Marital status |  |  |  |
| Unmarried (Ref.) |  |  |  |
| Married | 0.78 (0.33 – 1.82) | 0.32 | 0.55 |
| Other | 0.39 (0.11 – 1.38) | 0.24 | 0.14 |
| Number of family members |  |  |  |
| 1-2 (Ref.) |  |  |  |
| 3-6 | 1.83 (1.03 – 3.22) | 0.50 | 0.03 |
| >6 | 1.66 (0.78 – 3.51) | 0.60 | 0.17 |

OR – Odds ratio (survey adjusted), SE – Standard error. CI – Confidence interval, Ref. – reference

**Table 21. Association of multi-dimension poverty with MDR (Model 11: Model 10 + family structure)**

| **Multi-dimension Poverty** | **aOR (95% CI)** | **robust SE** | **P-value** |
| --- | --- | --- | --- |
| Not deprived (Ref.) |  |  |  |
| Vulnerable | 2.20 (1.22 – 4.01) | 0.64 | 0.01 |
| Deprived | 2.26 (1.10 – 4.65) | 0.79 | 0.02 |
| Severely deprived | 3.36 (1.74 – 6.50) | 1.08 | 0.001 |
| Self-medication | 0.87 (0.49 – 1.53) | 0.24 | 0.62 |
| Course completion | 0.61 (0.26 – 1.41) | 0.24 | 0.23 |
| Dose skipping | 0.78 (0.34 – 1.76) | 0.31 | 0.54 |
| WASH practices | 1.45 (0.87 – 2.42) | 0.36 | 0.14 |
| Gender |  |  |  |
| Male (ref.) |  |  |  |
| Female | 0.81 (0.58 – 1.14) | 0.13 | 0.22 |
| Age |  |  |  |
| 18 – 30 (Ref.) |  |  |  |
| 31 – 40 | 0.88 (0.44 – 1.76) | 0.29 | 0.71 |
| 41 – 50 | 0.82 (0.35 – 1.90) | 0.33 | 0.64 |
| 51 – 60 | 0.47 (0.22 – 1.01) | 0.17 | 0.05 |
| >60 | 0.53 (0.22 – 1.24) | 0.22 | 0.13 |
| Marital status |  |  |  |
| Unmarried (Ref.) |  |  |  |
| Married | 0.80 (0.35 – 1.81) | 0.32 | 0.59 |
| Other | 0.41 (0.12 – 1.38) | 0.24 | 0.14 |
| Number of family members |  |  |  |
| 1-2 (Ref.) |  |  |  |
| 3-6 | 1.94 (1.08 – 1.48) | 0.55 | 0.02 |
| >6 | 1.94 (0.86 – 4.35) | 0.76 | 0.10 |
| Family structure |  |  |  |
| Nuclear (Ref.) |  |  |  |
| Joint/extended | 0.79 (0.47 – 1.33) | 0.20 | 0.37 |

OR – Odds ratio (survey adjusted), SE – Standard error. CI – Confidence interval, Ref. – reference

**Table 22. Association of multi-dimension poverty with MDR (Model 12: Model 11 + education)**

| **Multi-dimension Poverty** | **aOR (95% CI)** | **robust SE** | **P-value** |
| --- | --- | --- | --- |
| Not deprived (Ref.) |  |  |  |
| Vulnerable | 3.10 (1.36 – 7.02) | 1.23 | 0.008 |
| Deprived | 3.43 (1.38 – 8.52) | 1.52 | 0.009 |
| Severely deprived | 5.21 (2.18 – 12.43) | 2.21 | 0.001 |
| Self-medication | 0.83 (0.48 – 1.45) | 0.22 | 0.51 |
| Course completion | 0.60 (0.26 – 1.38) | 0.24 | 0.22 |
| Dose skipping | 0.81 (0.35 – 1.86) | 0.32 | 0.61 |
| WASH practices | 1.45 (0.87 – 2.41) | 0.36 | 0.14 |
| Gender |  |  |  |
| Male (ref.) |  |  |  |
| Female | 0.85 (0.62 – 1.18) | 0.36 | 0.34 |
| Age |  |  |  |
| 18 – 30 (Ref.) |  |  |  |
| 31 – 40 | 0.93 (0.45 – 1.90) | 0.32 | 0.83 |
| 41 – 50 | 0.82 (0.35 – 1.93) | 0.34 | 0.65 |
| 51 – 60 | 0.47 (0.22 – 1.01) | 0.17 | 0.05 |
| >60 | 0.54 (0.22 – 1.29) | 0.23 | 0.16 |
| Marital status |  |  |  |
| Unmarried (Ref.) |  |  |  |
| Married | 0.81 (0.35 – 1.86) | 0.33 | 0.61 |
| Other | 0.39 (0.11 – 1.37) | 0.24 | 0.14 |
| Number of family members |  |  |  |
| 1-2 (Ref.) |  |  |  |
| 3-6 | 1.89 (1.06 – 3.36) | 0.53 | 0.03 |
| >6 | 1.96 (0.87 – 4.39) | 0.77 | 0.09 |
| Family structure |  |  |  |
| Nuclear (Ref.) |  |  |  |
| Joint/extended | 0.84 (0.50 – 1.39) | 0.20 | 0.48 |
| Education |  |  |  |
| No education (Ref.) |  |  |  |
| Primary | 1.15 (0.71 – 1.84) | 0.26 | 0.54 |
| Secondary | 1.85 (0.88 – 3.92) | 0.67 | 0.10 |
| Other | 1.72 (0.73 – 4.02) | 0.71 | 0.20 |

OR – Odds ratio (survey adjusted), SE – Standard error. CI – Confidence interval, Ref. – reference

**Table 23. Association of multi-dimension poverty with MDR (Model 13: Model 12 + employment)**

| **Multi-dimension Poverty** | **aOR (95% CI)** | **robust SE** | **P-value** |
| --- | --- | --- | --- |
| Not deprived (Ref.) |  |  |  |
| Vulnerable | 3.04 (1.35 – 7.06) | 1.24 | 0.009 |
| Deprived | 3.41 (1.37 – 8.42) | 1.52 | 0.01 |
| Severely deprived | 5.15 (2.16 – 12.29) | 2.18 | 0.001 |
| Self-medication | 0.85 (0.48 – 1.52) | 0.24 | 0.59 |
| Course completion | 0.60 (0.26 – 1.39) | 0.24 | 0.23 |
| Dose skipping | 0.80 (0.35 – 1.83) | 0.32 | 0.59 |
| WASH practices | 1.44 (0.86 – 2.41) | 0.36 | 0.15 |
| Gender |  |  |  |
| Male (ref.) |  |  |  |
| Female | 0.83 (0.58 – 1.19) | 0.14 | 0.31 |
| Age |  |  |  |
| 18 – 30 (Ref.) |  |  |  |
| 31 – 40 | 0.92 (0.44 – 1.91) | 0.32 | 0.82 |
| 41 – 50 | 0.83 (0.35 – 1.95) | 0.34 | 0.66 |
| 51 – 60 | 0.46 (0.22 – 0.99) | 0.17 | 0.04 |
| >60 | 0.52 (0.21 – 1.29) | 0.23 | 0.15 |
| Marital status |  |  |  |
| Unmarried (Ref.) |  |  |  |
| Married | 0.85 (0.35 – 2.05) | 0.36 | 0.71 |
| Other | 0.42 (0.11 – 1.52) | 0.26 | 0.17 |
| Number of family members |  |  |  |
| 1-2 (Ref.) |  |  |  |
| 3-6 | 1.86 (1.05 – 3.29) | 0.51 | 0.03 |
| >6 | 1.94 (0.87 – 4.30) | 0.75 | 0.09 |
| Family structure |  |  |  |
| Nuclear (Ref.) |  |  |  |
| Joint/extended | 0.83 (0.50 – 1.37) | 0.20 | 0.47 |
| Education |  |  |  |
| No education (Ref.) |  |  |  |
| Primary | 1.16 (0.72 – 1.86) | 0.26 | 0.51 |
| Secondary | 1.96 (0.90 – 4.28) | 0.74 | 0.08 |
| Other | 1.84 (0.78 – 4.34) | 0.77 | 0.15 |
| Employment |  |  |  |
| Not employed (Ref.) |  |  |  |
| Employed | 0.87 (0.56 – 1.34) | 0.18 | 0.52 |

OR – Odds ratio (survey adjusted), SE – Standard error. CI – Confidence interval, Ref. – reference

**Table 24. Association of multi-dimension poverty with MDR (Model 14: Model 13 +ability to meet healthcare cost)**

| **Multi-dimension Poverty** | **aOR (95% CI)** | **robust SE** | **P-value** |
| --- | --- | --- | --- |
| Not deprived (Ref.) |  |  |  |
| Vulnerable | 3.16 (1.42 – 7.09) | 1.23 | 0.006 |
| Deprived | 3.35 (1.33 – 8.42) | 1.51 | 0.01 |
| Severely deprived | 4.90 (1.96 – 12.22) | 2.18 | 0.001 |
| Self-medication | 0.83 (0.47 – 1.47) | 0.23 | 0.53 |
| Course completion | 0.61 (0.26 – 1.42) | 0.25 | 0.25 |
| Dose skipping | 0.79 (0.34 – 1.81) | 0.32 | 0.57 |
| WASH practices | 1.48 (0.86 – 2.52) | 0.38 | 0.14 |
| Gender |  |  |  |
| Male (ref.) |  |  |  |
| Female | 0.84 (0.58 – 1.22) | 0.15 | 0.36 |
| Age |  |  |  |
| 18 – 30 (Ref.) |  |  |  |
| 31 – 40 | 0.92 (0.44 – 1.92) | 0.33 | 0.83 |
| 41 – 50 | 0.81 (0.35 – 1.89) | 0.33 | 0.62 |
| 51 – 60 | 0.47 (0.21 – 1.03) | 0.18 | 0.06 |
| >60 | 0.52 (0.20 – 1.33) | 0.23 | 0.17 |
| Marital status |  |  |  |
| Unmarried (Ref.) |  |  |  |
| Married | 0.84 (0.35 – 2.02) | 0.36 | 0.69 |
| Other | 0.42 (0.11 – 1.52) | 0.26 | 0.18 |
| Number of family members |  |  |  |
| 1-2 (Ref.) |  |  |  |
| 3-6 | 1.82 (0.92 – 3.58) | 0.60 | 0.08 |
| >6 | 1.84 (0.77 – 4.40) | 0.78 | 0.16 |
| Family structure |  |  |  |
| Nuclear (Ref.) |  |  |  |
| Joint/extended | 0.82 (0.51 – 1.31) | 0.18 | 0.40 |
| Education |  |  |  |
| No education (Ref.) |  |  |  |
| Primary | 1.19 (0.75 – 1.89) | 0.26 | 0.44 |
| Secondary | 2.04 (0.96 – 4.32) | 0.74 | 0.06 |
| Other | 1.92 (0.84 – 4.35) | 0.76 | 0.11 |
| Employment |  |  |  |
| Not employed (Ref.) |  |  |  |
| Employed | 0.88 (0.56 – 1.37) | 0.19 | 0.58 |
| Ability to meet healthcare cost |  |  |  |
| Easy (Ref.) |  |  |  |
| Little difficult | 1.11 (0.61 – 2.03) | 0.32 | 0.70 |
| Very difficult | 1.31 (0.68 – 2.53) | 0.42 | 0.39 |

OR – Odds ratio (survey adjusted), SE – Standard error. CI – Confidence interval, Ref. – reference

**Table 25. Association of multi-dimension poverty with MDR (Model 15: Model 14 +UTI history)**

| **Multi-dimension Poverty** | **aOR (95% CI)** | **robust SE** | **P-value** |
| --- | --- | --- | --- |
| Not deprived (Ref.) |  |  |  |
| Vulnerable | 3.17 (1.43 – 7.01) | 1.23 | 0.006 |
| Deprived | 3.36 (1.34 – 8.40) | 1.50 | 0.01 |
| Severely deprived | 4.94 (1.97 – 12.36) | 2.21 | 0.001 |
| Self-medication | 0.85 (0.48 – 1.51) | 0.23 | 0.57 |
| Course completion | 0.61 (0.26 – 1.43) | 0.25 | 0.25 |
| Dose skipping | 0.80 (0.35 – 1.84) | 0.32 | 0.59 |
| WASH practices | 1.46 (0.88 – 2.45) | 0.36 | 0.13 |
| Gender |  |  |  |
| Male (ref.) |  |  |  |
| Female | 0.85 (0.59 – 1.22) | 0.14 | 0.37 |
| Age |  |  |  |
| 18 – 30 (Ref.) |  |  |  |
| 31 – 40 | 0.93 (0.45 – 1.93) | 0.33 | 0.85 |
| 41 – 50 | 0.82 (0.35 – 1.89) | 0.33 | 0.64 |
| 51 – 60 | 0.48 (0.22 – 1.05) | 0.18 | 0.06 |
| >60 | 0.53 (0.21 – 1.33) | 0.23 | 0.17 |
| Marital status |  |  |  |
| Unmarried (Ref.) |  |  |  |
| Married | 0.84 (0.35 – 2.05) | 0.36 | 0.70 |
| Other | 0.42 (0.11 – 1.50) | 0.26 | 0.17 |
| Number of family members |  |  |  |
| 1-2 (Ref.) |  |  |  |
| 3-6 | 1.81 (0.92 – 3.58) | 0.60 | 0.08 |
| >6 | 1.82 (0.76 – 4.34) | 0.77 | 0.16 |
| Family structure |  |  |  |
| Nuclear (Ref.) |  |  |  |
| Joint/extended | 0.83 (0.51 – 1.37) | 0.20 | 0.47 |
| Education |  |  |  |
| No education (Ref.) |  |  |  |
| Primary | 1.20 (0.72 – 2.01) | 0.30 | 0.45 |
| Secondary | 2.08 (0.95 – 4.54) | 0.79 | 0.06 |
| Other | 1.93 (0.85 – 4.38) | 0.77 | 0.10 |
| Employment |  |  |  |
| Not employed (Ref.) |  |  |  |
| Employed | 0.88 (0.55 – 1.39) | 0.19 | 0.57 |
| Ability to meet healthcare cost |  |  |  |
| Easy (Ref.) |  |  |  |
| Little difficult | 1.13 (0.61 – 2.08) | 0.33 | 0.68 |
| Very difficult | 1.35 (0.67 – 2.69) | 0.45 | 0.37 |
| UTI history |  |  |  |
| No (Ref.) |  |  |  |
| Yes | 0.91 (0.48 – 1.73) | 0.28 | 0.77 |

OR – Odds ratio (survey adjusted), SE – Standard error. CI – Confidence interval, Ref. – reference

**Table 26. Association of multi-dimension poverty with MDR (Model 16: Model 15 + symptoms stigma)**

| **Multi-dimension Poverty** | **aOR (95% CI)** | **robust SE** | **P-value** |
| --- | --- | --- | --- |
| Not deprived (Ref.) |  |  |  |
| Vulnerable | 3.01 (1.33 – 6.84) | 1.20 | 0.01 |
| Deprived | 3.17 (1.26 – 7.96) | 1.42 | 0.01 |
| Severely deprived | 4.55 (1.79 – 11.56) | 2.07 | 0.002 |
| Self-medication | 0.81 (0.45 – 1.46) | 0.23 | 0.49 |
| Course completion | 0.61 (0.26 – 1.42) | 0.25 | 0.24 |
| Dose skipping | 0.81 (0.35 – 1.88) | 0.33 | 0.62 |
| WASH practices | 1.49 (0.89 – 2.50) | 0.37 | 0.11 |
| Gender |  |  |  |
| Male (ref.) |  |  |  |
| Female | 0.75 (0.50 – 1.13) | 0.14 | 0.16 |
| Age |  |  |  |
| 18 – 30 (Ref.) |  |  |  |
| 31 – 40 | 0.94 (0.45 – 1.94) | 0.33 | 0.86 |
| 41 – 50 | 0.84 (0.36 – 1.94) | 0.34 | 0.67 |
| 51 – 60 | 0.51 (0.23 – 1.15) | 0.20 | 0.10 |
| >60 | 0.56 (0.21 – 1.44) | 0.26 | 0.22 |
| Marital status |  |  |  |
| Unmarried (Ref.) |  |  |  |
| Married | 0.84 (0.34 – 2.04) | 0.36 | 0.70 |
| Other | 0.42 (0.12 – 1.49) | 0.26 | 0.17 |
| Number of family members |  |  |  |
| 1-2 (Ref.) |  |  |  |
| 3-6 | 1.85 (0.94 – 3.62) | 0.60 | 0.06 |
| >6 | 1.92 (0.79 – 4.64) | 0.82 | 0.13 |
| Family structure |  |  |  |
| Nuclear (Ref.) |  |  |  |
| Joint/extended | 0.81 (0.48 – 1.36) | 0.20 | 0.42 |
| Education |  |  |  |
| No education (Ref.) |  |  |  |
| Primary | 1.22 (0.73 – 2.05) | 0.30 | 0.42 |
| Secondary | 2.07 (0.96 – 4.44) | 0.77 | 0.06 |
| Other | 1.90 (0.86 – 4.20) | 0.73 | 0.10 |
| Employment |  |  |  |
| Not employed (Ref.) |  |  |  |
| Employed | 0.89 (0.55 – 1.41) | 0.20 | 0.61 |
| Ability to meet healthcare cost |  |  |  |
| Easy (Ref.) |  |  |  |
| Little difficult | 1.11 (0.60 – 2.05) | 0.33 | 0.71 |
| Very difficult | 1.29 (0.65 – 2.55) | 0.43 | 0.44 |
| UTI history |  |  |  |
| No (Ref.) |  |  |  |
| Yes | 0.84 (0.43 – 1.63) | 0.27 | 0.60 |
| Symptoms stigma |  |  |  |
| No (Ref.) |  |  |  |
| Yes | 1.37 (0.74 – 2.54) | 0.41 | 0.29 |

OR – Odds ratio (survey adjusted), SE – Standard error. CI – Confidence interval, Ref. – reference

**Table 27. Association of multi-dimension poverty with MDR (Model 17: full covariate)**

| **Multi-dimension Poverty** | **aOR (95% CI)** | **robust SE** | **P-value** |
| --- | --- | --- | --- |
| Not deprived (Ref.) |  |  |  |
| Vulnerable | 3.03 (1.33 – 6.73) | 1.18 | 0.009 |
| Deprived | 3.01 (1.26 – 7.15) | 1.27 | 0.01 |
| Severely deprived | 4.28 (1.74 – 10.49) | 1.87 | 0.002 |
| Self-medication | 0.82 (0.45 – 1.46) | 0.23 | 0.49 |
| Course completion | 0.64 (0.27 – 1.48) | 0.26 | 0.29 |
| Dose skipping | 0.82 (0.36 – 1.88) | 0.33 | 0.64 |
| WASH practices | 1.51 (0.90 – 2.53) | 0.38 | 0.10 |
| Gender |  |  |  |
| Male (ref.) |  |  |  |
| Female | 0.77 (0.51 – 1.16) | 0.15 | 0.21 |
| Age |  |  |  |
| 18 – 30 (Ref.) |  |  |  |
| 31 – 40 | 0.92 (0.44 – 1.90) | 0.32 | 0.81 |
| 41 – 50 | 0.83 (0.36 – 1.95) | 0.34 | 0.67 |
| 51 – 60 | 0.51 (0.23 – 1.16) | 0.20 | 0.10 |
| >60 | 0.57 (0.22 – 1.50) | 0.27 | 0.25 |
| Marital status |  |  |  |
| Unmarried (Ref.) |  |  |  |
| Married | 0.85 (0.35 – 2.06) | 0.36 | 0.72 |
| Other | 0.42 (0.12 – 1.48) | 0.26 | 0.17 |
| Number of family members |  |  |  |
| 1-2 (Ref.) |  |  |  |
| 3-6 | 1.85 (0.94 – 3.64) | 0.61 | 0.07 |
| >6 | 1.87 (0.75 – 4.64) | 0.83 | 0.16 |
| Family structure |  |  |  |
| Nuclear (Ref.) |  |  |  |
| Joint/extended | 0.82 (0.49 – 1.36) | 0.20 | 0.44 |
| Education |  |  |  |
| No education (Ref.) |  |  |  |
| Primary | 1.24 (0.73 – 2.11) | 0.32 | 0.40 |
| Secondary | 2.13 (0.96 – 4.72) | 0.82 | 0.05 |
| Other | 1.96 (0.87 – 4.38) | 0.77 | 0.09 |
| Employment |  |  |  |
| Not employed (Ref.) |  |  |  |
| Employed | 0.89 (0.55 – 1.42) | 0.20 | 0.61 |
| Ability to meet healthcare cost |  |  |  |
| Easy (Ref.) |  |  |  |
| Little difficult | 1.10 (0.60 – 2.01) | 0.32 | 0.74 |
| Very difficult | 1.22 (0.61 – 2.42) | 0.40 | 0.54 |
| UTI history |  |  |  |
| No (Ref.) |  |  |  |
| Yes | 0.81 (0.41 – 1.60) | 0.26 | 0.54 |
| Symptoms stigma |  |  |  |
| No (Ref.) |  |  |  |
| Yes | 1.36 (0.74 – 2.51) | 0.40 | 0.30 |
| Healthcare advice |  |  |  |
| Formal (Ref.) |  |  |  |
| Informal | 1.23 (0.67 – 2.27) | 0.36 | 0.71 |

OR – Odds ratio (survey adjusted), SE – Standard error. CI – Confidence interval, Ref. – reference

**Table 28. Association between multi-dimension poverty and MDR, adjusted for individual covariates in survey-weighted logistic regression models**

|  | **Adjusted for antibiotic misuse** | | |
| --- | --- | --- | --- |
| **Multi-dimension Poverty** | aOR (95% CI) | robust SE | P-value |
| Not deprived (Ref.) | - | - | - |
| Vulnerable | 2.01 (1.13 – 3.56) | 0.56 | 0.01 |
| Deprived | 2.02 (1.06 – 3.87) | 0.64 | 0.03 |
| Severely deprived | 1.74 (1.02 – 2.93) | 0.44 | 0.03 |
| **Self-medication** |  |  |  |
| No (Ref.) | - | - | - |
| Yes | 0.91(0.52 – 1.59) | 0.25 | 0.74 |
| **Course completion** |  |  |  |
| No (Ref.) | - | - | - |
| Yes | 0.63(0.27 – 1.47) | 0.26 | 0.28 |
| **Dose skipping** |  |  |  |
| No (Ref.) | - | - | - |
| Yes | 0.72 (0.31 – 1.64) | 0.29 | 0.42 |
|  | **Adjusted for WASH practices** | | |
| Not deprived (Ref.) | - | - | - |
| Vulnerable | 2.02 (1.16 – 3.50) | 0.54 | 0.01 |
| Deprived | 2.36 (1.16 – 4.80) | 0.81 | 0.01 |
| Severely deprived | 2.25 (1.16 – 4.39) | 0.73 | 0.01 |
| **WASH Practices** |  |  |  |
| Poor (Ref.) | - | - | - |
| Good | 1.37 (0.81 – 2.32) | 0.35 | 0.03 |
|  | **Adjusted for gender** | | |
| Not deprived (Ref.) | - | - | - |
| Vulnerable | 2.04 (1.14 – 3.63) | 0.57 | 0.01 |
| Deprived | 2.09 (1.06 – 4.10) | 0.68 | 0.03 |
| Severely deprived | 1.84 (1.05 – 3.21) | 0.50 | 0.03 |
| **Gender** |  |  |  |
| Male (Ref.) | - | - | - |
| Female | 0.84 (0.59 – 1.19) | 0.14 | 0.33 |
|  | **Adjusted for age** | | |
| Not deprived (Ref.) | - | - | - |
| Vulnerable | 2.01 (1.14 – 3.55) | 0.55 | 0.01 |
| Deprived | 2.19 (1.12 – 4.25) | 0.71 | 0.22 |
| Severely deprived | 2.64 (1.52 – 4.57) | 0.70 | 0.001 |
| **Age (years)** |  |  |  |
| 18 – 30 (Ref.) | - | - | - |
| 31 – 40 | 0.82 (0.53 – 1.28) | 0.17 | 0.37 |
| 41 – 50 | 0.84 (0.46 – 1.52) | 0.24 | 0.56 |
| 51 – 60 | 0.46 (0.27 – 0.78) | 0.11 | 0.006 |
| >60 | 0.45 (0.25 – 0.83) | 0.13 | 0.01 |
|  | **Adjusted for marital status** | | |
| Not deprived (Ref.) | - | - | - |
| Vulnerable | 2.06 (1.17 – 3.62) | 0.56 | 0.01 |
| Deprived | 2.13 (1.09 – 4.12) | 0.68 | 0.02 |
| Severely deprived | 2.18 (1.22 – 3.90) | 0.61 | 0.01 |
| **Marital status** |  |  |  |
| Unmarried (Ref.) | - | - | - |
| Married | 0.74 (0.44 – 1.26) | 0.19 | 0.26 |
| Other | 0.30 (0.11 – 0.77) | 0.13 | 0.01 |
|  | **Adjusted for family structure** | | |
| Not deprived (Ref.) | - | - | - |
| Vulnerable | 1.99 (1.12 – 3.51) | 0.55 | 0.01 |
| Deprived | 2.12 (1.10 – 4.08) | 0.67 | 0.02 |
| Severely deprived | 1.88 (0.97 – 3.65) | 0.60 | 0.05 |
| **Family structure** |  |  |  |
| Nuclear (Ref.) | - | - | - |
| Joint/extended | 0.93 (0.56 – 1.54) | 0.22 | 0.78 |
|  | **Adjusted for number of family members** | | |
| Not deprived (Ref.) | - | - | - |
| Vulnerable | 1.85 (1.03 – 3.31) | 0.52 | 0.03 |
| Deprived | 1.86 (0.98 – 3.55) | 0.58 | 0.05 |
| Severely deprived | 1.70 (0.91 – 3.17) | 0.51 | 0.08 |
| **Number of family members** |  |  |  |
| 1-2 (Ref.) | - | - | - |
| 3-6 | 1.60 (0.92 – 2.77) | 0.43 | 0.08 |
| >6 | 1.34 (0.69 – 2.57) | 0.42 | 0.36 |
|  | **Adjusted for education** | | |
| Not deprived (Ref.) | - | - | - |
| Vulnerable | 2.59 (1.01 – 6.62) | 1.18 | 0.04 |
| Deprived | 2.91 (1.02 – 8.27) | 1.48 | 0.04 |
| Severely deprived | 2.73 (1.04 – 7.19) | 1.22 | 0.04 |
| **Education** |  |  |  |
| No education (Ref.) | - | - | - |
| Primary | 1.40 (0.90 – 2.17) | 0.30 | 0.12 |
| Secondary | 1.95 (0.93 – 4.10) | 0.70 | 0.07 |
| Other | 1.61 (0.70 – 3.69) | 0.65 | 0.25 |
|  | **Adjusted for employment** | | |
| Not deprived (Ref.) | - | - | - |
| Vulnerable | 1.89 (1.04 – 3.43) | 0.55 | 0.03 |
| Deprived | 1.98 (1.01 – 3.92) | 0.66 | 0.04 |
| Severely deprived | 1.73 (1.01 – 2.95) | 0.45 | 0.04 |
| **Employment** |  |  |  |
| Not employed (Ref.) | - | - | - |
| Employed | 0.92 (0.65 – 1.29) | 0.15 | 0.06 |
|  | **Adjusted for ability to meet healthcare cost** | | |
| Not deprived (Ref.) | - | - | - |
| Vulnerable | 1.86 (1.04 – 3.32) | 0.52 | 0.03 |
| Deprived | 1.87 (0.92 – 3.78) | 0.64 | 0.07 |
| Severely deprived | 1.57 (0.75 – 3.28) | 0.56 | 0.22 |
| **Ability to meet healthcare cost** |  |  |  |
| Easy (Ref.) | - | - | - |
| Little difficult | 1.15 (0.68 – 1.93) | 0.29 | 0.58 |
| Very difficult | 1.26 (0.69 – 2.30) | 0.37 | 0.42 |
|  | **Adjusted for UTI history** | | |
| Not deprived (Ref.) | - | - | - |
| Vulnerable | 1.98 (1.15 – 3.41) | 0.52 | 0.01 |
| Deprived | 2.12 (1.10 – 4.09) | 0.68 | 0.02 |
| Severely deprived | 1.90 (1.02 – 3.52) | 0.57 | 0.04 |
| **UTI history** |  |  |  |
| No (Ref.) | - | - | - |
| Yes | 0.90 (0.55 – 1.47) | 0.21 | 0.69 |
|  | **Adjusted for symptom stigma** | | |
| Not deprived (Ref.) | - | - | - |
| Vulnerable | 1.84 (1.00 – 3.38) | 0.54 | 0.04 |
| Deprived | 1.94 (0.95 – 3.97) | 0.67 | 0.06 |
| Severely deprived | 1.66 (0.87 – 3.14) | 0.51 | 0.11 |
| **Symptom stigma** |  |  |  |
| No (Ref.) | - | - | - |
| Yes | 1.19 (0.71 – 2.01) | 0.30 | 0.48 |
|  | **Adjusted for healthcare advice** | | |
| Not deprived (Ref.) | - | - | - |
| Vulnerable | 1.86 (1.06 – 3.25) | 0.50 | 0.03 |
| Deprived | 1.82 (0.96 – 3.44) | 0.56 | 0.06 |
| Severely deprived | 1.54 (0.82 – 2.86) | 0.46 | 0.16 |
|  | **Adjusted for healthcare advice** | | |
| **Healthcare advice** |  |  |  |
| Formal (Ref.) | - | - | - |
| Informal | 1.22 (0.78 – 1.91) | 0.26 | 0.34 |

OR – Odds ratio (survey adjusted), SE – Standard error. CI – Confidence interval, Ref. – reference

## **Association of antibiotic misuse, WASH practices and demographic variables with MDR**

**Table 29. Association of antibiotic misuse indicators, wash practices and demographic variables with MDR**

|  | **Antibiotic misuse** | | |
| --- | --- | --- | --- |
|  | OR (95% CI) | robust SE | P-value |
| **Self-medication** |  |  |  |
| No (Ref.) | - | - | - |
| Yes | 1.26 (0.85 – 1.85) | 0.23 | 0.22 |
| **Course completion** |  |  |  |
| No (Ref.) | - | - | - |
| Yes | 0.73 (0.47 – 1.12) | 0.15 | 0.15 |
| **Dose skipping** |  |  |  |
| No (Ref.) |  |  |  |
| Yes | 1.26 (0.83 – 1.91) | 0.25 | 0.24 |
|  | **WASH Practices** | | |
| **WASH Practices** |  |  |  |
| Poor (Ref.) | - | - | - |
| Good | 0.86 (0.56 – 1.31) | 0.17 | 0.48 |
|  | **Gender** | | |
| **Gender** |  |  |  |
| Male (Ref.) |  |  |  |
| Female | 0.95 (0.69 – 1.30) | 0.14 | 0.76 |
|  | **Age** | | |
| **Age (years)** |  |  |  |
| 18 – 30 (Ref.) | - | - | - |
| 31 – 40 | 0.90 (0.57 – 1.43) | 0.20 | 0.67 |
| 41 – 50 | 1.14 (0.63 – 2.07) | 0.33 | 0.64 |
| 51 – 60 | 0.72 (0.41 – 1.28) | 0.20 | 0.26 |
| >60 | 0.86 (0.45 – 1.65) | 0.27 | 0.65 |
|  | **Marital status** | | |
| **Marital status** |  |  |  |
| Unmarried (Ref.) | - | - | - |
| Married | 0.92 (0.57 – 1.48) | 0.21 | 0.73 |
| Other | 0.51 (0.20 – 1.32) | 0.23 | 0.16 |
|  | **Family structure** | | |
| **Family structure** |  |  |  |
| Nuclear (Ref.) | - | - | - |
| Joint/extended | 1.32 (0.88 – 2.01) | 0.26 | 0.16 |
|  | **Number of family members** | | |
| **Number of family members** |  |  |  |
| 1-2 (Ref.) | - | - | - |
| 3-6 | 1.85 (1.09 – 3.13) | 0.47 | 0.02 |
| >6 | 1.95 (1.09 – 3.48) | 0.55 | 0.02 |
|  | **Education** | | |
| **Education** |  |  |  |
| No education (Ref.) | - | - | - |
| Primary | 1.36 (0.91 – 2.02) | 0.26 | 0.12 |
| Secondary | 0.84 (0.51 – 1.38) | 0.20 | 0.48 |
| Other | 0.63 (0.43 – 0.93) | 0.11 | 0.02 |
|  | **Employment** | | |
| **Employment** |  |  |  |
| Not employed (Ref.) | - | - | - |
| Employed | 0.70 (0.48 – 1.01) | 0.12 | 0.05 |
|  | **Ability to meet healthcare cost** | | |
| **Ability to meet healthcare cost** |  |  |  |
| Easy (Ref.) | - | - | - |
| Little difficult | 1.41 (0.84 – 2.35) | 0.35 | 0.17 |
| Very difficult | 1.79 (1.13 – 2.82) | 0.39 | 0.01 |
|  | **UTI history** | | |
| **UTI history** |  |  |  |
| No (Ref.) | - | - | - |
| Yes | 1.20 (0.78 – 1.83 | 0.24 | 0.38 |
|  | **Symptom stigma** | | |
| **Symptom stigma** |  |  |  |
| No (Ref.) | - | - | - |
| Yes | 1.49 (0.98 – 2.26) | 0.30 | 0.06 |
|  | **Healthcare advice** | | |
| **Healthcare advice** |  |  |  |
| Formal (Ref.) | - | - | - |
| Informal | 1.57 (1.04 – 2.39) | 0.32 | 0.03 |

OR – Odds ratio (survey adjusted), SE – Standard error. CI – Confidence interval, Ref. – reference

## **Association of multi-dimension poverty with antibiotic misuse indicators**

**Table 30. Association of multi-dimension poverty with antibiotic misuse indicators**

|  | **Self-medication** | | |
| --- | --- | --- | --- |
| MPI | OR (95% CI) | robust SE | P-value |
| Not deprived (Ref.) | - | - | - |
| Vulnerable | 3.65 (1.95 – 6.83) | 1.11 | <0.001 |
| Deprived | 3.53 (2.02 – 6.16) | 0.96 | <0.001 |
| Severely deprived | 10.80 (5.75 – 20.28) | 3.32 | <0.001 |
|  | **Course completion** | | |
| Not deprived (Ref.) | - | - | - |
| Vulnerable | 0.53 (0.30 – 0.93) | 0.14 | 0.03 |
| Deprived | 0.27 (0.15 – 0.50) | 0.08 | <0.001 |
| Severely deprived | 0.07 (0.04 – 0.12) | 0.02 | <0.001 |
|  | **Dose skipping** | | |
| Not deprived (Ref.) | - | - | - |
| Vulnerable | 2.37 (1.26 – 4.48) | 0.73 | 0.009 |
| Deprived | 3.37 (1.85 – 6.15) | 0.99 | <0.001 |
| Severely deprived | 13.5 (7.54 – 24.17) | 3.84 | <0.001 |

OR – Odds ratio (survey adjusted), SE – Standard error. CI – Confidence interval, Ref. – reference

## **Association of multi-dimension poverty with WASH practices**

**Table 31. Association of multi-dimension poverty with WASH practices**

|  | **WASH Practices** | | |
| --- | --- | --- | --- |
| MPI | OR (95% CI) | robust SE | P-value |
| Not deprived (Ref.) | - | - | - |
| Vulnerable | 0.26 (0.13 – 0.52) | 0.08 | <0.001 |
| Deprived | 0.06 (0.02 – 0.14) | 0.02 | <0.001 |
| Severely deprived | 0.01 (0.01 – 0.03) | 0.005 | <0.001 |

OR – Odds ratio (survey adjusted), SE – Standard error. CI – Confidence interval, Ref. – reference

**References**

1. Green DL, Keenan K, Fredricks KJ, et al. The role of multidimensional poverty in antibiotic misuse: a mixed-methods study of self-medication and non-adherence in Kenya, Tanzania, and Uganda. *The Lancet Global Health* 2023; **11**(1): e59-e68.

2. Fransman T, Yu D. Multidimensional poverty in South Africa in 2001–16. *Development Southern Africa* 2019; **36**(1): 50-79.

3. Zafar U, Iqbal A, Basit A, et al. Antibiotic Prescribing Practices in a Tertiary Care Teaching Hospital: A Retrospective Cross-Sectional Analysis. *Cureus* 2024; **16**(11): e73092.

4. DRAP. GUIDELINES ON RESPONSIBLE USE OF ANTIMICROBIALS IN HUMAN HEALTH 2021. <https://www.dra.gov.pk/wp-content/uploads/2022/02/Guidelines-Responsible-Use-of-Antimicrobials-1.pdf> (accessed Dec-24 2024).

5. Khan Z, Khan J, Khan A, et al. Prescribing pattern of antibiotics and guideline adherence in acute community-acquired uncomplicated urinary tract infections in Pakistani women. *Journal of Health Sciences and Medicine* 2019.
